# *Panax notoginseng* flower protects against diabetic cardiomyopathy by regulating the ACSL4/ALOX15 pathway

**DOI:** 10.3389/fphar.2026.1780442

**Published:** 2026-03-27

**Authors:** Yue Zhou, Limin Ouyang, Linyue Dong, Xue Zhang, Jun Du, Shuai Sun, John R. Ussher, Jordan S. F. Chan, Liang Chen, Doudou Huang, Yiming Li

**Affiliations:** 1 Department of TCM Chemistry, School of Pharmacy, Shanghai University of Traditional Chinese Medicine, Shanghai, China; 2 Healthy Aging Research Center, Amway (Shanghai) Innovation and Science Co., Ltd., Shanghai, China; 3 Faculty of Pharmacy and Pharmaceutical Sciences, University of Alberta, Edmonton, AB, Canada; 4 Alberta Diabetes Institute, University of Alberta, Edmonton, AB, Canada; 5 Cardiovascular Research Institute, University of Alberta, Edmonton, AB, Canada

**Keywords:** ACSL4/ALOX15, diabetic cardiomyopathy, ferroptosis, *Panax notoginseng* flower, PGC-1 alpha

## Abstract

**Background:**

*Panax notoginseng* (Burk.) F. H. Chen flower (SQH), a common traditional Chinese medicine and edible food, has long been shown to protect against diabetes. However, the protective effects of SQH against diabetic cardiomyopathy (DbCM) and its potential mechanisms are not yet understood.

**Aim of the Study:**

This study aimed to analyze the therapeutic effects of the flowers of *Panax notoginseng* (SQH) on DbCM and elucidate its molecular mechanisms.

**Materials and Methods:**

The *in vitro* model of DbCM was established using palmitate-treated H9c2 cardiomyocyte. A high-fat diet (HFD) in conjunction with streptozotocin (STZ)-induced DbCM mouse model was utilized to validate the cardioprotective effects and mechanism of SQH. Transcriptomic analysis was used to examine the potential mechanisms, followed by both *in vitro* and *in vivo* validation of key protein expression levels in the ACSL4/ALOX15 signaling pathway.

**Results:**

*In vitro*, SQH treatment significantly protected H9c2 cells from palmitic acid (PA)-induced injury by reducing lipid peroxidation and improving mitochondrial membrane function. Transcriptomic data indicated that SQH influenced ferroptosis-related pathways. Moreover, SQH suppressed the ACSL4/ALOX15 pathway, leading to decreased levels of the key lipid peroxidation product 12-HETE and upregulation of GPX4. In DbCM mice, SQH administration improved glucose homeostasis, attenuated cardiac dysfunction, and reduced myocardial lipid peroxidation.

**Conclusion:**

This study demonstrated that SQH ameliorated DbCM injury primarily by inhibiting ACSL4/ALOX15-mediated ferroptosis. These findings not only highlight *Panax notoginseng* flower as a promising therapeutic candidate for the early intervention of DbCM but also reveal the underlying mechanism of SQH’s protective effect.

## 1 Introduction

Diabetic cardiomyopathy (DbCM) is clinically defined as left ventricular dysfunction occurring in diabetic patients, independent of other risk factors or cardiovascular diseases (Seferović et al., 2024), which is usually characterized by an increase in the cross-sectional area of cardiomyocytes, a reduction in the e'/a' ratio and E/A ratio, and an increase in the E/e' ratio (Ritchie and Abel, 2020). The prevalence of DbCM can reach approximately 67% among diabetic individuals according to the least restrictive diagnostic criteria (Segar et al., 2021). DbCM is a multifactorial disease, and the complicated factors, such as increased circulating fatty acids and hyperglycemia, ferroptosis, ROS, ER stress, and changed Ca^2+^ handling, alter phenotypes within the cardiomyocyte (Bugger and Abel, 2014; Wang and Hill, 2015; Nakamura and Sadoshima, 2020). Oxidative stress is a key factor arising from lipid accumulation and increased plasma free fatty acid levels in the obese heart (van de Weijer et al., 2011). The increased ROS induces further mitochondrial dysfunction and energy metabolism disorder, resulting in fibrosis, diastolic dysfunction, and heart failure in diabetic individuals (Jia et al., 2018). Iron overload, reduced cystine uptake, and glutathione depletion may trigger ferroptosis and contribute to the development of DbCM (Tian et al., 2024). However, the precise mechanisms underlying DbCM remain poorly understood, which continues to hinder the translation of preclinical findings into clinical applications. Although numerous preclinical studies have indicated potential therapeutic benefits of various drugs for DbCM, none have been approved by the FDA for clinical treatment of this condition (Lee et al., 2021; Wilding et al., 2021). Therefore, there is an urgent need to develop effective therapeutic interventions for DbCM.

*Panax notoginseng* (Burk.) F. H. Chen has been commonly used in traditional Chinese medicine (TCM) in China for thousands of years and has been shown to protect against diabetes and diabetic cardiomyopathy (Yang et al., 2010; Fan et al., 2016; Zhang et al., 2022). Research indicates that the cardioprotective effects of *Panax notoginseng* are primarily mediated by its saponins, which confer protection against ferroptosis through the activation of the Nrf2 pathway (Du et al., 2021). The dried flowers of *P. notoginseng* (SQH), known as a medicine–food homologous resource, have the highest abundance of saponins compared to the fruit pedicel and fruit pericarp (Ma et al., 2021). Additionally, SQH demonstrates efficacy in preventing arrhythmia and mitigating myocardial ischemia (Tian, 2000; Zheng et al., 2010). However, the efficacy of SQH on DbCM remains unknown, and its underlying mechanisms of action have yet to be elucidated.

Ferroptosis, a form of programmed cell death induced by lethal lipid peroxidation, plays an important role in the pathogenesis of DbCM (Stockwell et al., 2017; Wu et al., 2021). Recent studies have shown that inhibition of ferroptosis may be a promising therapeutic strategy for the treatment of DbCM. Empagliflozin ameliorated DbCM-induced cardiac remodeling and dysfunction by alleviating ferroptosis through the USP7/NRF2 pathway (Cui et al., 2025). Walnut peptides alleviated ferroptosis by activating the CD36 pathway and promoting glutathione synthesis, thereby improving myocardial injury (Xiang et al., 2025). In brief, targeting ferroptosis represents a novel therapy for DbCM.

In this study, we aimed to systematically investigate the therapeutic potential of SQH on DbCM and its underlying mechanisms. H9c2 cardiomyocytes treated with palmitate (0.2 mM) were utilized to assess the cardioprotective effects of SQH, and transcriptomic analysis was used to examine the potential mechanisms. A high-fat diet (HFD) in conjunction with a streptozotocin (STZ)-induced DbCM mouse model was used to validate the cardioprotective effects and underlying mechanism of SQH. This study provides a theoretical scientific basis for the treatment of DbCM with SQH.

## 2 Materials and methods

### 2.1 Reagents and chemicals

*Panax notoginseng* flower was purchased from Guangdong Yifang Pharmaceutical Co., Ltd. (batch number SY23080401). Compounds baimaside, ginsenoside Ra_2_, ginsenoside Rb_1_, ginsenoside Rc, ginsenoside Ra_1_, ginsenoside Rb_2_, ginsenoside Rb_3_, and ginsenoside Re were purchased from Chengdu Aibock Biotechnology Co., Ltd. (Sichuan, China). The Cell Counting Kit-8 (MA0218), BCA Protein Assay Kit (MA0082), and dichlorofluorescein diacetate (DCFH-DA) reagents (MB4682) were purchased from Meilunbio. The Total Superoxide Dismutase (SOD) Assay Kit with WST-8(S0101S), GSH and GSSG Assay Kit (S0053), ATP Assay Kit (S0026), Antifade Mounting with DAPI (P0131), and Mitochondrial Membrane Potential Assay Kit with TMRE (C2001S) were obtained from Beyotime. The MDA Assay Kit (G4300) and Annexin V-FITC/PI Cell Apoptosis Detection Kit (G1511) were purchased from Servicebio. Lipid Peroxidation Probe-BDP 581/591 C11 (L267) and MitoPeDPP were obtained from Dojindo Chemistry. The EZ-press RNA Purification Kit was purchased from EZB (B0004D).

### 2.2 Sample preparation

Dried *Panax notoginseng* flower (100 g) was ground and subjected to two rounds of reflux extraction (2 h each) using a 10-fold volume of 70% aqueous ethanol. The extract was filtered and then concentrated under vacuum at 50 °C until completely dry to obtain 48.5 g of solid residue (SQH), which was stored at 4 °C.

### 2.3 UHPLC–qTOF–MS/MS analysis

SQH (20.0 mg) was dissolved in 70% methanol (10 mL) and centrifuged at 10,000 rpm for 20 min, and the supernatants were prepared for chemical analysis. Chemical profiling of SQH was conducted on Q-TOF MS (6530B, Agilent Technologies) equipped with a 1290 UHPLC separation system; the injection volume was 2 μL. A Waters ACQUITY UPLC BEH C18 column (2.1 mm × 100 mm; 1.7 µm) was used. A mobile phase consisting of two solvents, namely, solvent A (aqueous formic acid, 0.1% v/v) and solvent B (acetonitrile acidified with 0.1% formic acid), at a flow rate of 0.3 mL/min was used. The optimized elution program was as follows: 0 min–5 min (5%–35% B), 5 min–12 min (35%–35% B), and 12 min–17 min (40%–95% B). The column temperature was maintained at 50 °C, while the auto-sampler was maintained at 4 °C. Mass spectra were recorded using ESI in the negative ionization mode with a capillary voltage of 3,500 V. The mass spectrometry conditions were set as follows: gas temperature, 350 °C; drying gas flow rate, 11 L/min; nebulization pressure, 45 psi; and mass spectra range of 100 m/z–1,700 m/z. The voltages of the skimmer and fragmentation were set at 65 and 140 V, respectively, with a collision energy of 30 V. Peaks and spectra were processed using MassHunter Workstation B.10.00 (Agilent Technologies) and were tentatively identified based on their mass spectra, exact masses, and fragmentation patterns, compared with previously reported data.

### 2.4 Cell culture and treatments

H9c2 cells were purchased from Dalian Meilunbio Biotechnology Co., Ltd. (Dalian, China). Dulbecco’s Modified Eagle’s Medium (DMEM), supplemented with 10% fetal bovine serum (FBS) (Meilunbio, China) and streptomycin (100 μg/mL), was used to culture H9c2 cells. Cells were maintained in a humidified incubator at 37 °C with 5% CO_2_ and sub-cultured in a 1:3 ratio every 3 days. When the cell density reached 50%, H9c2 cells were pretreated with SQH (100 or 200 μg/mL) or PBS for 2 h. Then, the medium was replaced with complete medium containing either BSA-conjugated 0.2 mM palmitic acid (PA) or vehicle control for 24 h.

### 2.5 Animal and treatment

The 9-week-old male C57BL/6J mice (Laboratory Animal Center, Shanghai University of T.C.M, Shanghai, China), weighing 24 g–28 g, were maintained in a temperature-controlled room under a 12-h light/ 12-h dark cycle. Each mouse received unlimited drinking water. The Laboratory Animal Committee of Shanghai University of T.C.M. approved all the animal experiments (No. PZSHUTCM2508030005).

A type 2 DbCM model was induced in mice by 12 weeks of HFD) (60% kcal from lard, D12492) feeding, followed by a single intraperitoneal injection of streptozotocin (STZ, 75 mg/kg) at week 4, as previously described (Almutairi et al., 2021). Two weeks post-STZ injection, 40 male C57BL/6J mice (DbCM and sham) were randomized into five groups: control, DbCM, metformin (250 mg/kg), SQH-L (200 mg/kg), and SQH-H (400 mg/kg), receiving daily oral gavage of PBS or drug until study termination.

### 2.6 Glucose and insulin tolerance testing

Mice were transferred to clean cages and fasted for 12 h with free access to drinking water prior to IP glucose tolerance and 4 h prior to IP insulin tolerance. All mice received a single dose of glucose (2 g/kg) or insulin (0.75 IU/kg) via IP injection. Blood glucose levels were measured from the tail vein at 0, 30, 60, 90, and 120 min with a glucose meter.

### 2.7 Ultrasound echocardiography

At 2 and 8 week post-STZ injection, mice were anesthetized with 2%–3% isoflurane to undergo echocardiography using a VisualSonics Vevo 2100 Rodent Ultrasound Imaging System. Several parameters were detected to analyze left ventricular (LV) systolic function and diastolic function, including LV ejection fraction, LV fractional shortening, E/A ratio, e’/a’ ratio, and E/e’ ratio.

### 2.8 Blood tests

On the last day of treatment, blood was collected from all mice to prepare serum samples. Lipid content, including triglycerides (TG), total cholesterol (TC), high-density lipoprotein cholesterol (HDL-C), and low-density lipoprotein cholesterol (LDL-C), was measured using an automatic biochemical analyzer. The lactate dehydrogenase (LDH) content, insulin levels, and SOD levels were quantified according to the manufacturer’s instructions in the test kits.

### 2.9 Histological examination

For histological studies, part of the heart tissu was formalin-fixed and paraffin-imbedded before heart slicing. The samples were sent to Hunan Aifang Biotechnology Co., Ltd. for hematoxylin–eosin staining (H&E), periodic acid–Schiff staining (PAS), picrosirius red staining (PSR), and wheat germ agglutinin (WGA) staining.

### 2.10 Cell viability

Cell viability was determined by the CCK-8 method. Rat cardiac myoblast H9c2 cells were seeded in 96-wells with a cell volume of 1 × 10^4^ per well. After removing the cell culture medium, 100 µl of diluted CCK-8 reagent (diluted 10 times with FBS-free cell medium) was added to each well. Then, the cells were incubated for 1 h. The absorbance of each well was measured at 450 nm using a microplate reader.

### 2.11 Cell apoptosis detection

Cell apoptosis was detected using an Annexin V-FITC/PI Cell Apoptosis Detection Kit. Cells were harvested and washed twice with cold PBS. Annexin V-FITC/PI probes were separately diluted with binding buffer in a 1:20 ratio. The cells were placed in a 37 °C incubator for 10 min in the dark. Before detection using a fluorescence microscope, four times the volume of binding buffer was used to dilute the supernatant. Fluorescence signals were observed using a fluorescence microscope with FITC and PI filters.

### 2.12 Intracellular ROS level

The intracellular ROS level was detected using the DCFH-DA probe (Meilunbio). The DCFH-DA probe was diluted to a final concentration of 10 μM in the culture medium and added to each group of cells. The cells were placed in a 37 °C incubator for 30 min in the dark. Then, the cells were washed twice with PBS to wash off the probe that had not entered the cells. The fluorescence intensity was quantified using a fluorescence microscope.

### 2.13 BODIPY fluorescence staining

The lipid peroxide radical level was detected using the BODIPY 581/591 C11 fluorescent probe. After the original cell culture medium was removed, the BODIPY probe was added to each group of cells in the culture medium at a dilution of 1 : 1,000. Then, the cells were stained in the dark for 30 min, and the probe that did not enter the cells was washed away with PBS. A fluorescence microscope was used to observe and image the fluorescence intensity.

### 2.14 Mitochondrial lipid peroxides level

The mitochondrial lipid peroxide level was measured using the MitoPeDPP fluorescent probe. H9c2 cells were cultured in 24-well plates and treated as mentioned. In brief, the stock solution of MitoPeDPP was diluted to 0.5 μM for staining in DMEM. Cells were washed twice with HBSS and incubated with MitoPeDPP for 30 min at 37 °C in the dark. Fluorescence signals were detected using a microscope at excitation/emission of 470/525 nm.

### 2.15 TMRE staining

Mitochondrial membrane potential was detected using the tetramethylrhodamine ethyl ester (TMRE) probe. The working solution was prepared by diluting TMRE 1000X to TMRE 0.1X with the cell culture medium at room temperature for 30 min in a dark room. The excess probe was washed off twice with PBS. The image and fluorescence intensity were observed using a fluorescence microscope.

### 2.16 SOD, ATP, MDA, and GSH levels

H9c2 cells were cultured in Petri dishes and pretreated with SQH (100 and 200 μg/mL) in the same way. The samples were collected at 24 h after adding PA and determined by methods based on the manufacturer’s instructions.

### 2.17 Determination of 12-HETE in cells

12-HETE was extracted from the supernatant by solid phase extraction using Elut C18 columns. The quantification of 12-HETE was analyzed using an ultra-high-performance liquid chromatography (UPLC) system (Thermo Fisher Scientific, UltiMate 3000) coupled with triple quadrupole mass spectrometry (Thermo Fisher Scientific, TSQ Quantum Access Max). 12-HETE was identified and quantified by MRM as previously described (Dong et al., 2024).

### 2.18 Quantitative real-time PCR

Total RNA was extracted using the EZ-press RNA Purification Kit (EZBioscience), and the cDNA was synthesized utilizing HiScript II Q RT SuperMix (Vazyme). Quantitative real-time PCR (qRT-PCR) analysis was conducted using the Universal SYBR Green Fast qPCR Mix (ABclonal). The relative gene expression was determined using the 2^−ΔΔCT^ method and normalized to the housekeeping gene β-actin. The ACSL4, ALOX15, and PGC-1α primers were designed by Generay (Shanghai, China).

### 2.19 Western blot analysis

Total protein was extracted from cells collected after treatment with PA for 24 h. Lysis buffer containing protease and phosphatase inhibitors was added to the samples, and the protein concentration was measured using a BCA assay (Beyotime, China). Thereafter, an appropriate amount of protein extract was added to SDS–polyacrylamide gel wells and transferred to polyvinylidene difluoride membrane (Sigma). The membrane was blocked with 5% skimmed milk in TBST at room temperature for 2 h and incubated with primary antibodies overnight at 4 °C. The membrane was washed once with TBST, followed by a 1:10,000 dilution of horseradish peroxidase-conjugated secondary antibody for 1 h. Protein bands were visualized using an enhanced chemiluminescence reagent (Meilunbio). Primary and secondary antibodies were acquired from the following commercial sources: anti-GPX4 and anti-ACSL4 antibody were purchased from Proteintech (10020795 and 00098000); anti-caspase 3 and anti-cleaved caspase 3 were purchased from ABclonal (A0214 and A19664). Anti-cytochrome c, anti-PGC-1α, and goat anti-rabbit IgG H&L (HRP) were purchased from Zen-Bio (R22867, L26SE02, and 511203). Anti-15 lipoxygenase was purchased from Abcam (ab244205). Anti-beta-actin antibody was purchased from Sharebio (SB-AB2001).

### 2.20 Transcriptomic study

Total RNA was extracted using the TRIzol reagent (Invitrogen, CA, United States) according to the manufacturer’s protocol. RNA purity and quantification were evaluated using the NanoDrop 2000 Spectrophotometer (Thermo Fisher Scientific, United States). RNA integrity was assessed using the Agilent 2100 Bioanalyzer (Agilent Technologies, Santa Clara, CA, United States). The libraries were constructed using the VAHTS Universal V6 RNA-seq Library Prep Kit, according to the manufacturer’s instructions. Transcriptome sequencing and analysis were conducted by OE Biotech Co., Ltd. (Shanghai, China).

According to the above sequencing results, differential expression analysis was performed using DESeq2^5^. A *p*-value < 0.05 and fold change > 2 or fold change < 0.5 were set as the threshold for significantly differentially expressed genes (DEGs). Hierarchical cluster analysis of DEGs was performed using R (v 3.2.0) to demonstrate the expression pattern of genes in different groups and samples.

### 2.21 Statistical analysis

Data are presented as the mean ± SEM. Furthermore, all data were analyzed using GraphPad Prism 8.0. ImageJ was used to analyze Western blot protein bands and fluorescence images. Differences were analyzed by two-tailed Student’s t-test or one-way ANOVA, followed by Dunnett’s multiple comparisons test. A *p* < 0.05 was considered statistically significant.

## 3 Results

### 3.1 LC–MS analysis of the major constituents in SQH

**FIGURE 1:**
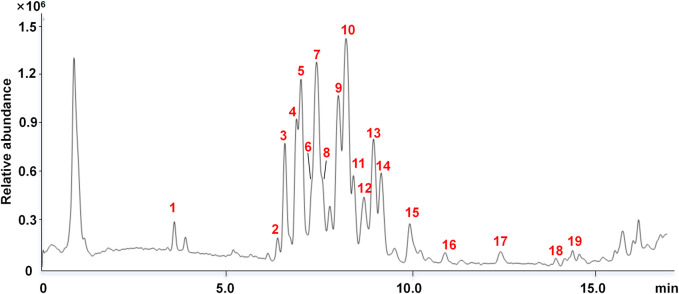
Total ion chromatography of UPLC–QTOF–MS/MS analysis of SQH in negative mode.

Chemical characterization of SQH was performed using LC–qTOF–MS/MS analysis in negative mode, leading to the tentative identification of 19 compounds, including 1 flavonoid and 18 saponins. Among them, baimaside, ginsenoside Ra_2_, ginsenoside Rb_1_, ginsenoside Rc, ginsenoside Ra_1_, ginsenoside Rb_2_, ginsenoside Rb_3_, and ginsenoside Re were identified by comparing their retention times and MS data with the reference standards. Other components were identified by comparing the MS/MS data with those of the references (Yang et al., 2013; Li et al., 2017). The details of the identified compounds are summarized in Table 1, and the total ion chromatography is illustrated in Figure 1. The mass error of the molecular ions for all identified compounds was within ± 5 ppm, indicating that the empirical molecular formulas were well-matched the putative molecular ions. All tentative compounds were verified based on their MS/MS spectra in comparison with the reported literature.

**TABLE 1:** Nineteen identified chemicals in SQH using LC–MS analysis.

Peak no.	Name	Formula	ms^1^	Rt (min)	Mass error (ppm)	ms^2^
1	Baimaside^a^	C_27_H_30_O_17_	625.1407	3.574	−0.52	300.0279 and 151.0041
2	PPD-20-glc-xyl-xyl-xyl-3-glc-glc	C_63_H_106_O_30_	1,341.6686	6.496	−0.76	1,239.6362, 805.9837, 716.336, and 693.3345
3	PPD-20-glc-glc-xyl-3-glc-glc	C_59_H_100_O_27_	1,239.6379	6.678	−0.02	805.9847, 693.3341, 665.3207, and 642.3188
4	Ginsenoside Ra_2_ ^a^	C_58_H_98_O_26_	1,209.6273	7.008	−0.05	805.9884, 693.3331, and 627.3120
5	Ginsenoside Rb_1_ ^a^	C_54_H_92_O_23_	1,107.5966	7.133	0.85	805.9886, 599.3004, and 576.2984
6	Ginsenoside Rc^a^	C_53_H_90_O_22_	1,077.5861	7.542	0.93	805.9890, 627.3118, 584.2949, and 561.2932
7	Ginsenoside Ra_1_ ^a^	C_58_H_98_O_26_	1,209.6275	7.553	0.12	1,077.5861, 805.9872, 627.3137, and 561.2919
8	PPD-20-glc-xyl-xyl-xyl-3-glc-glc isomer	C_63_H_106_O_30_	1,341.6697	7.712	0.06	1,077.5832, 693.3338, and 670.3320
9	Ginsenoside Rb_2_ ^a^	C_53_H_90_O_22_	1,077.5854	8.156	0.28	965.9998, 805.9837, 584.2943, and 561.2916
10	Ginsenoside Rb_3_ ^a^	C_53_H_90_O_22_	1,077.5867	8.372	1.49	805.9813, 584.2945, and 561.2927
11	Ginsenoside Rg_7_	C_58_H_98_O_26_	1,209.6284	8.605	0.86	1,077.5819, 650.3167, 627.3138, and 604.3112
12	PPD-glc-xyl-glc-glc-AcO	C_55_H_92_O_23_	1,119.5955	8.872	−0.15	805.9861 and 582.2976
13	Ginsenoside Rs_1_ or Rs_2_	C_55_H_92_O_23_	1,119.5973	9.156	1.46	805.9872 and 582.2980
14	PPD-20-AcO-glc-xyl-3-glc-glc	C_55_H_92_O_23_	1,119.5960	9.372	0.3	805.9862 and 582.2959
15	Ginsenoside Re^a^	C_48_H_82_O_18_	945.5437	10.174	0.91	805.9831 and 505.2492
16	Isomer of ginsenoside Re	C_48_H_82_O_18_	945.5438	12.67	1.01	805.9886 and 582.7963
17	Notoginsenoside O	C_52_H_88_O_21_	1,047.5758	13.687	1.21	805.9898 and 243.8990
18	PPD-20-glc-3-glc-xyl	C_47_H_80_O_17_	915.5340	14.051	1.88	783.4894 and 621.4388
19	Isomer of PPD-20-glc-3-glc-xyl	C_47_H_80_O_17_	915.5342	14.483	2.1	783.4906 and 621.4379

^a^ Compounds were identified with authentic standards.

The tentatively identified compounds are tabulated in Table 1 with their chromatographic and HRMS data, providing the phytochemical profiling of SQH.

### 3.2 SQH protected H9c2 cells against PA-induced oxidative stress

**FIGURE 2:**
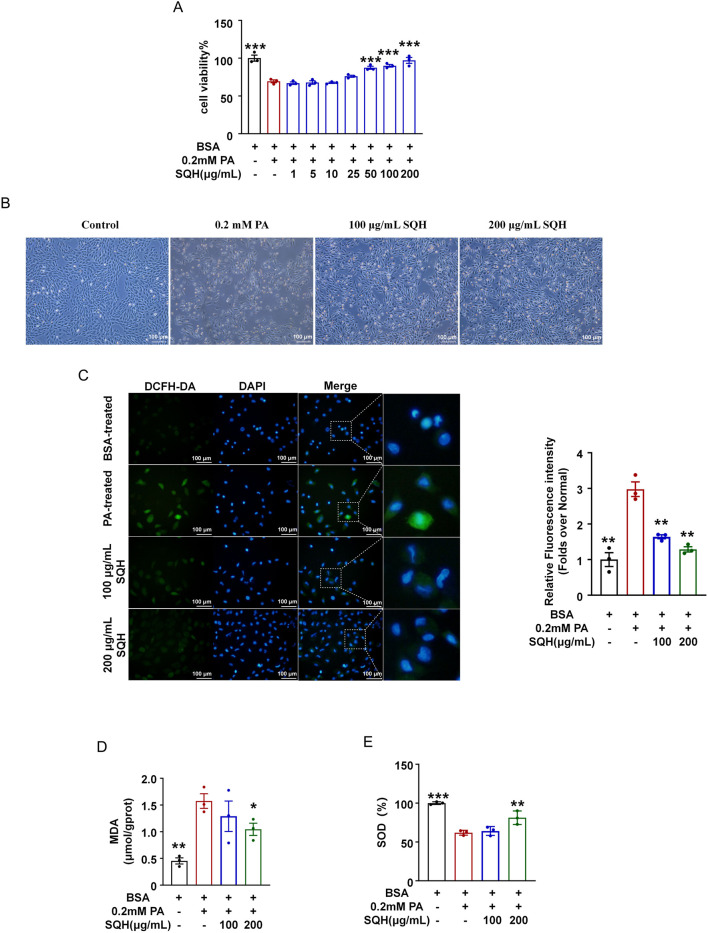
SQH protected H9c2 cells against PA-induced oxidative stress. **(A)** Cell viability under different PA concentrations. **(B)** Cell viability under 0.2 mM PA with SQH treatment (bar = 100 μm). **(C)** Fluorescence staining of ROS (green) in H9c2 cells treated with SQH (100 and 200 μg/mL) or PBS (bar = 100 μm). The relative fluorescence intensity was analyzed using the ratio of green fluorescence intensity to the number of total cells in three randomly selected areas. **(D)** MDA level in H9c2 cells treated with SQH (100 and 200 μg/mL) or PBS. **(E)** SOD enzyme activity in H9c2 cells treated with SQH (100 and 200 μg/mL) or PBS. *p < 0.05, **p < 0.01, and ***p < 0.001 vs. PA (+) group.

H9c2 cells were treated with 0.2 mM PA for 24 h to induce a lipotoxicity model. Cell viability was reduced to 69.63% with 0.2 mM PA treatment, while SQH significantly increased the cell viability at 50 μg/mL–200 μg/mL concentration (Figures 2A,B). Oxidative stress, a critical driver of diabetic cardiomyopathy, arises from an imbalance between ROS production and antioxidant capacity (Jia et al., 2018). We found that the total ROS levels, detected using the DCFH-DA probe, were significantly increased in PA-induced cells, and this increase was concentration-dependently mitigated by SQH treatment (Figure 2C). SQH (200 μg/mL) also markedly decreased the content of MDA and restored the activity of the antioxidant enzyme SOD (Figures 2D,E). Taken together, these results indicated that SQH could suppress lipotoxicity-induced ROS accumulation in myocardial cells.

### 3.3 The protective effect of SQH was potentially mediated by the inhibition of ferroptosis

**FIGURE 3:**
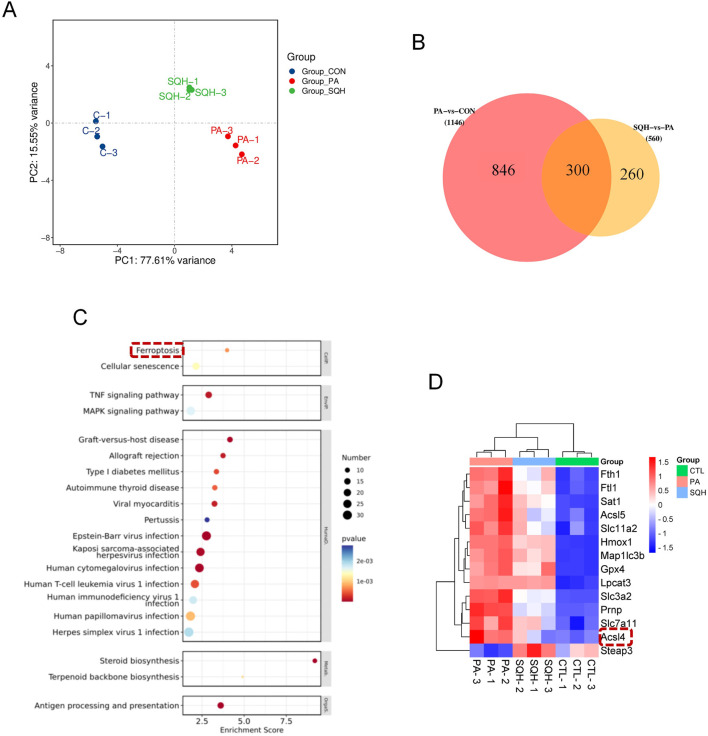
Protective effect of SQH was potentially mediated by the inhibition of ferroptosis. **(A)** PCA between the three groups (CON, PA, and SQH). **(B)** Venn diagram of differentially expressed genes. **(C)** KEGG enrichment of the top 20 signaling pathway. **(D)** Summary heatmap of quantitative RNA-seq analysis of genes related with ferroptosis in H9c2 cells treated with 100 μg/mL SQH or PBS. *p < 0.05, **p < 0.01, and ***p < 0.001 vs. PA (+) group.

To identify key signaling pathways in the lipotoxicity model, transcriptomic profiles of three groups were compared. Principal component analysis (PCA) revealed clear separation among the three groups (Figures 3A,B), indicating distinct transcriptomic landscapes. KEGG pathway enrichment analysis highlighted ferroptosis as the most significantly altered pathway (Figure 3C). Key ferroptosis-related genes, including upstream regulators *Acsl4* and *Sat1*, were markedly altered (Figure 3D), although *Alox15* was not detected due to its low abundance. RNA-seq indicated that SQH attenuated PA-induced cardiomyocyte injury by modulating ferroptosis.

### 3.4 SQH treatment inhibited ferroptosis by alleviating lipid peroxidation

**FIGURE 4:**
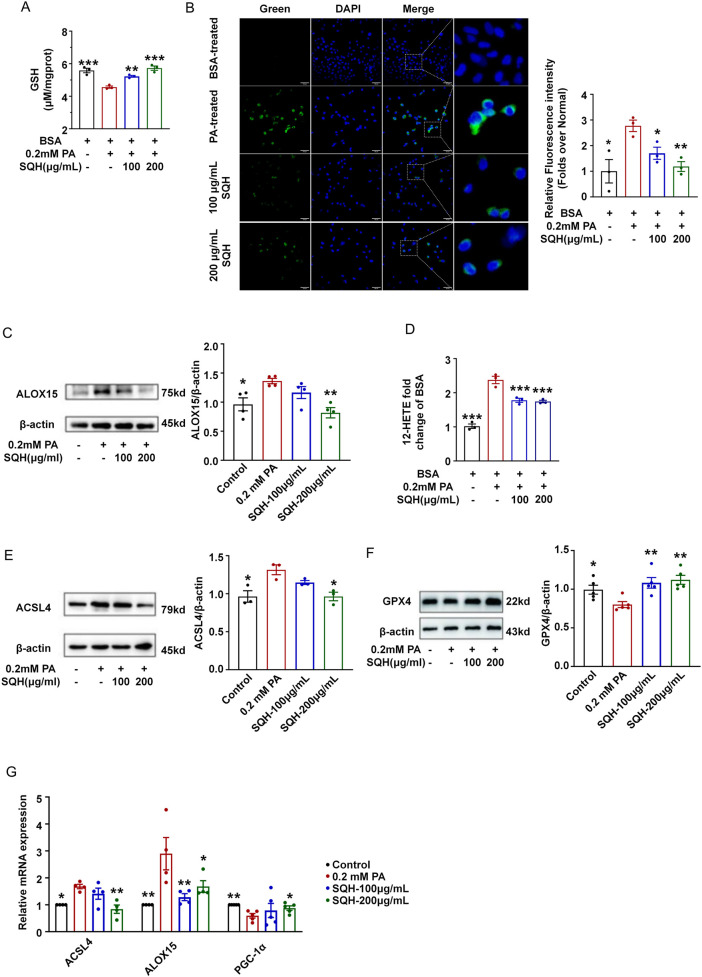
SQH treatment inhibited ferroptosis by alleviating lipid peroxidation. **(A)** GSH level in H9c2 cells treated with SQH (100 and 200 μg/mL) or PBS. **(B)** Fluorescence staining of BODIPY (green) in H9c2 cells treated with SQH (100 and 200 μg/mL) or PBS (bar = 100 μm). The relative fluorescence intensity was analyzed using the ratio of green fluorescence intensity to the number of total cells in three randomly selected areas. **(C)** Protein expressions for ALOX15 in SQH-treated H9c2 cells. **(D)** 12-HETE level of H9c2 cells under 0.2 mM PA. **(E)** Protein expressions for ALOX15 in SQH-treated H9c2 cells. **(F)** Protein expressions for GPX4 in SQH-treated H9c2 cells. **(G)** Quantitative RT-PCR analysis of genes *Alox15*, *Acsl4*, and *Pgc1a*. *p < 0.05, **p < 0.01, and ***p < 0.001 vs. PA (+) group.

To elucidate the role of SQH in ferroptosis inhibition, we assessed glutathione (GSH) content and lipid peroxidation in cells. The PA-induced GSH depletion was partially reversed by SQH (Figure 4A). Furthermore, green fluorescence in the PA group, indicating pronounced lipid peroxidation, was notably attenuated by SQH pre-treatment (Figure 4B). Results indicated that the expression and product level (12-HETE) of ALOX15 were significantly increased after PA induction, while SQH downregulated the protein content and activity (Figures 4C,D). Then, we analyzed the upstream protein ACSL4, given its established role in catalyzing the lipid peroxidation cascade (Yang et al., 2016; Doll et al., 2017; Stockwell et al., 2017; Cai et al., 2023). The upregulation of ACSL4 in the model was significantly suppressed by SQH (Figure 4E). A ferroptosis-related marker and the downstream protein GPX4 were decreased after PA treatment and restored to normal with 2 h of SQH pretreatment (Figure 4F). The mRNA level of ACSL4/ALXO15 was consistent with the protein expression trend (Figure 4G). These data collectively indicated that SQH mitigated lipid peroxidation by targeting the ferroptosis-related ACSL4/ALOX15 pathway signaling.

### 3.5 SQH protected mitochondrial function through PGC-1α

**FIGURE 5:**
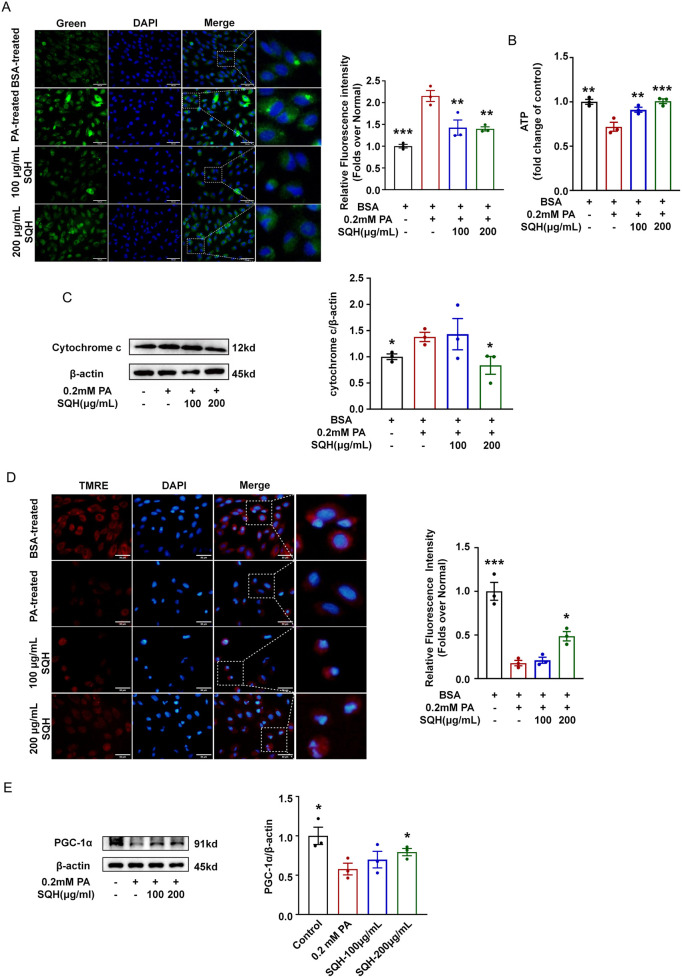
SQH protected mitochondrial function through PGC-1α. **(A)** Fluorescence staining of MitoPeDPP in H9c2 cells treated with SQH (100 and 200 μg/mL) or PBS (bar = 100 μm). The relative fluorescence intensity was analyzed using the ratio of green fluorescence intensity to the number of total cells in three randomly selected areas. **(B)** ATP levels in H9c2 cells treated with SQH (100 and 200 μg/mL) or PBS. **(C)** Western blots of Cyto c in H9c2 cells. **(D)** Fluorescence staining of TMRE (red) treated with SQH (100 and 200 μg/mL) or PBS. The relative fluorescence intensity was analyzed using the ratio of red fluorescence intensity to the number of total cells in three randomly selected areas. **(E)** Protein expressions for PGC-1α in SQH-treated H9c2 cells. *p < 0.05, **p < 0.01, and ***p < 0.001 vs. PA (+) group.

Mitochondrial dysfunction, characterized by excessive ROS and lipid peroxidation, is a critical event in ferroptosis (Chen et al., 2023). PA treatment triggered lipid peroxidation on the mitochondrial membrane, as shown in Figure 5A. Thus, ATP levels were significantly reduced, and Cyt c release was significantly increased by PA (Figures 5B,C). Therefore, mitochondrial membrane potential (MMP) was diminished in injured cardiomyocytes (Makrecka-Kuka et al., 2020). SQH treatment significantly reduced the lipid peroxidation level in the mitochondria and preserved mitochondrial respiratory function. SQH also concentration-dependently inhibited Cyt c release into the cytoplasm and improved MMP (Figures 5C,D), underscoring its role in maintaining mitochondrial integrity through inhibiting lipid peroxidation in mitochondria.

PGC-1α is a master regulator of mitochondrial biogenesis and respiration, and its expression level is a key determinant of cardiac oxidative capacity and mitochondrial content (Ventura-Clapier et al., 2008). Given prior evidence that ALOX15 promotes the degradation of PGC-1α and exacerbates ferroptosis (Cai et al., 2023), we assessed whether SQH could modulate PGC-1α expression. Our results showed that SQH treatment partially restored the PA-induced suppression of both PGC-1α protein and *Pgc1a* mRNA levels (Figures 5E, 4G). Taken together, these findings demonstrate that SQH protects H9c2 cells against PA-induced ferroptosis through the suppression of ACSL4/ALOX15 signaling and subsequent PGC-1α upregulation.

### 3.6 SQH intervention improves glucose homeostasis, serum lipids, and cardiac dysfunction in DbCM mice

**FIGURE 6:**
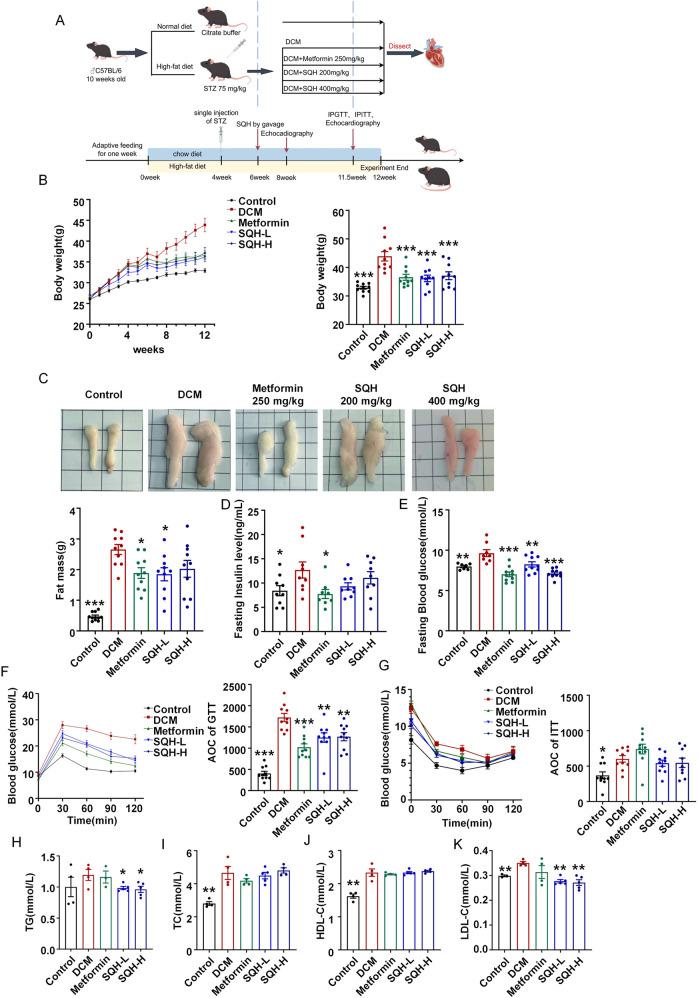
SQH intervention improves glucose homeostasis and serum lipids in DbCM mice. **(A)** Schematic diagram depicting the experimental strategy for DbCM models. **(B)** Body weight gain of the mice in 12 weeks (left), and body weights of the mice at 12 weeks of age (right). **(C)** Weight of adipose tissue around epididymis. **(D)** Serum insulin level at 12 weeks. **(E)** Fasting blood glucose level at 12 weeks. **(F)** Glucose tolerance and associated AOC at 12 weeks. **(G)** Insulin tolerance and associated AOC at 12 weeks. **(H)** Serum TG levels. **(I)** Serum TC levels. **(J)** Serum HDL-C levels. **(K)** Serum LDL-C levels in different groups. *p < 0.05, **p < 0.01, and ***p < 0.001 vs. DbCM group.

A T2D mouse model was established to analyze the therapeutic effect of SQH in DbCM (Figure 6A). SQH treatment could significantly attenuate the increased body weight in T2D mice (Figure 6B). This amelioration of body weight gain was associated with a reduction in epididymal adipose tissue mass (Figure 6C). SQH administration modestly improved fasting serum insulin levels and fasting blood glucose (Figures 6D,E), indicating improvement in insulin resistance. We further assessed glucose homeostasis via IPGTT and IPITT. Results indicated that both metformin and SQH improved glucose tolerance (Figure 6F). However, SQH showed a limited effect on overall insulin sensitivity according to the ITT results (Figure 6G). Next, we examined the impact of SQH on systemic metabolic alterations by serum lipid profiles. SQH treatment significantly lowered serum LDL-C levels and moderately attenuated the elevation of TG, while TC and HDL-C levels showed no significant changes in DbCM mice (Figures 6H–K).

**FIGURE 7:**
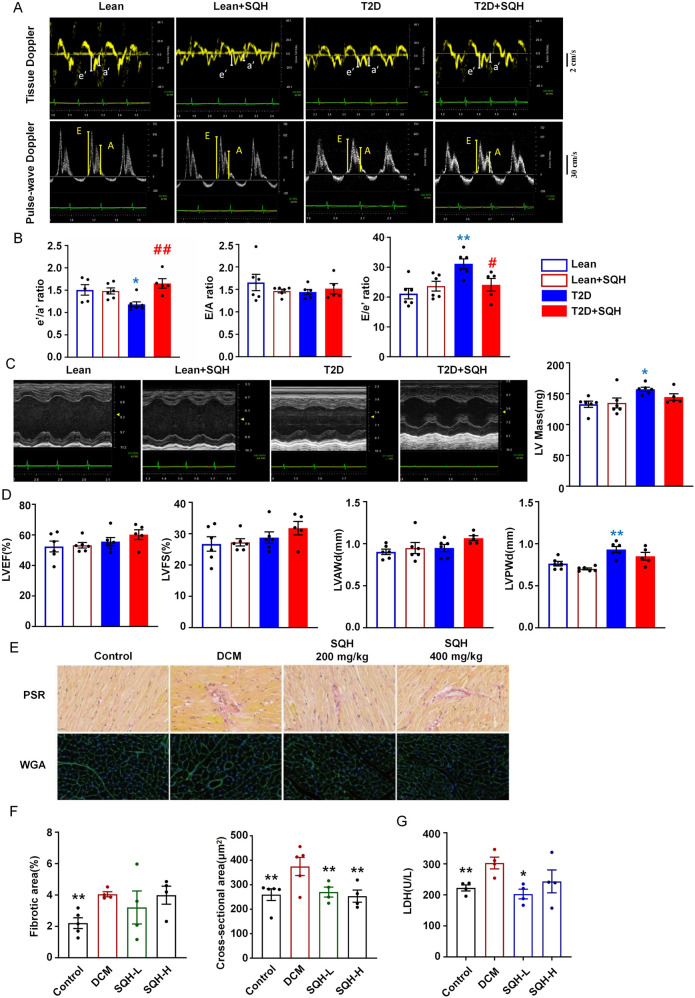
SQH intervention attenuates cardiac dysfunction in mice with T2D. **(A)** Representative images of Tissue Doppler and Pulse-wave Doppler in C57BL/6J mice with DbCM. **(B)** Tissue Doppler e’/a’ ratio, mitral E/A ratio, and E/e’ ratio assessed by ultrasound echocardiography. **(C)** Representative M-mode images and LV mass. **(D)** Left ventricular ejection fraction (LVEF), left ventricular fractional shortening (LVFS), left ventricular anterior wall diastole (LVAWd), and left ventricular posterior wall diastole (LVPWd) at end-systole were assessed via echocardiography. **(E)** Representative images of PSR-stained and WGA-stained sections. **(F)** Quantification of the cardiomyocyte fibrotic area and cross-sectional area based on PSR and WGA staining. **(G)** Serum levels of myocardial cell death marker LDH in DbCM and SQH-treated mice. *p < 0.05, **p < 0.01, and ***p < 0.001 vs. the DbCM group.

Based on the suboptimal efficacy of the high-dose group compared to that of the low-dose, we, hence, evaluated the cardiac function of the latter by echocardiographic Doppler (Supplementary Figure S1A). Consistent with previous studies (Ritchie and Abel, 2020; Gopal et al., 2021), our model mice exhibited significant diastolic dysfunction (Figures 7A,B). SQH effectively restored the e'/a' and E/e' ratios in DbCM mice, indicating a beneficial effect in diastolic function, despite the absence of an effect on the E/A ratio. However, we observed no changes in the systolic function parameters such as LVEF and LVFS, although the LV mass and LV posterior wall thickness increased in T2D mice (Figures 7C,D).

To assess the impact of SQH on myocardial structure in DbCM mice, we performed PSR and WGA staining to evaluate collagen deposition and cardiomyocyte cross-sectional area. Importantly, SQH treatment effectively decreased abnormal cardiomyocyte size in T2D mice (Figures 7E,F), indicating improvement in cardiomyocyte hypertrophy. However, treatment with SQH did not impact myocardial fibrosis. Furthermore, the serum LDH level decreased in the low-dose SQH group mice, indicating attenuated cardiac damage compared with that in the DbCM group (Figure 7G).

### 3.7 SQH protected against experimental diabetic cardiomyopathy by the ACSL4/ALOX15/PGC-1α signaling pathway

**FIGURE 8:**
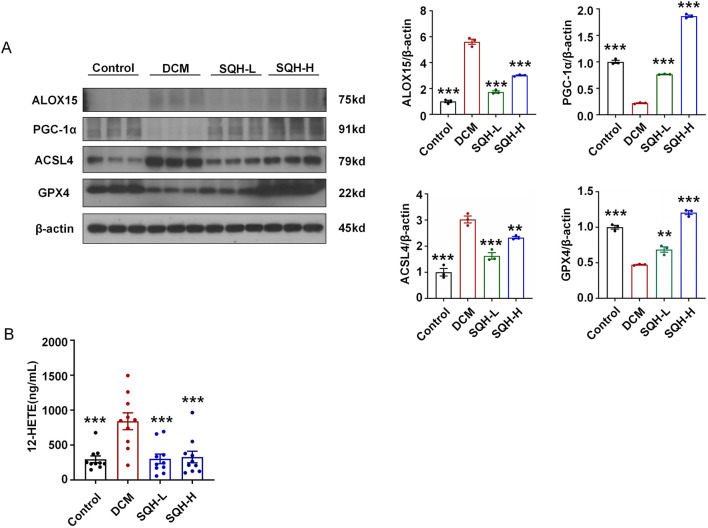
SQH reduced serum 12-HETE levels and protected against experimental DbCM by the ACSL4/ALOX15/PGC-1α signaling pathway. **(A)** Protein expressions for ALOX15, ACSL4, GPX4, and PGC-1α in DbCM mice heart tissues. **(B)** Serum 12-HETE levels in DbCM mice with or without SQH treatment.

**FIGURE 9:**
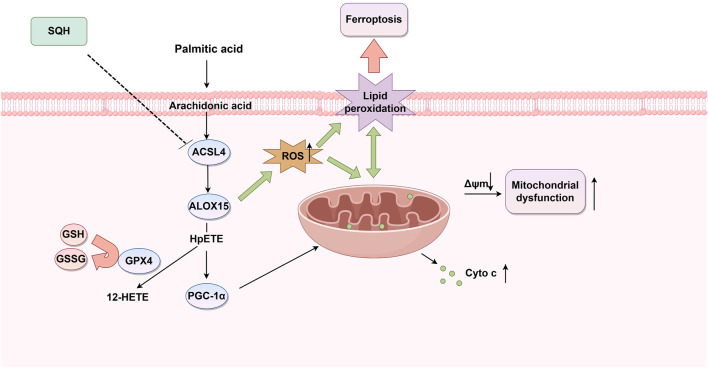
Schematic illustration showing the mechanisms underlying the prevention of PA-induced injury in cardiomyocytes by ACSL4/ALOX15/PGC-1α signaling. *Panax notoginseng* flower extract can ameliorate elevated ROS levels, lipid peroxidation, and mitochondrial dysfunction by regulating ACSL4/ALOX15/PGC-1α, thereby improving cardiac function and glucose homeostasis.

To validate our *in vitro* findings that the ACSL4/ALOX15 pathway is a potential therapeutic target for DbCM, we examined the expression of key proteins in mouse heart tissues. Results indicated that the protein levels of ACSL4 and ALOX15 were significantly upregulated in the model group and were suppressed by SQH treatment (Figure 8A), indicating an inhibition of the ACSL4/ALOX15 signaling pathway *in vivo*. The increased 12-HETE level was also alleviated by SQH (Figure 8B). Furthermore, SQH treatment robustly increased the protein level of GPX4 and PGC-1α. Collectively, these data revealed that SQH protected against experimental DbCM by modulating the ACSL4/ALOX15/PGC-1α signaling pathway (Figure 9).

## 4 Discussion

Our study reveals that the cardioprotective effect of SQH in DbCM is mediated through the inhibition of the ACSL4/ALOX15 signaling axis, thereby attenuating cardiomyocyte ferroptosis. This finding broadens our understanding of the pharmacological effects of SQH and links its therapeutic action to the ferroptosis pathway in DbCM. Previous studies have found that SQH and its active components exhibit potential cardioprotective effects against arrhythmia and myocardial ischemia by inhibiting cardiomyocyte apoptosis. Zhou et al. (2019) demonstrated that *P. notoginseng* flower extract promotes the growth of micro-vessels to improve the infarcted myocardium by upregulating HIF-1α, VEGFA, and KDR while concurrently inhibiting apoptosis via the Bax/Bcl-2 pathway, indicating therapeutic potential for cardiac repair. Notoginsenoside R1 protected H9c2 cardiomyocytes from apoptosis and hypertrophy induced by high glucose concentration through marked activation of AMPK/NRF2 signaling (Du et al., 2021). However, these studies have focused on cardiac injury models not related to diabetes. Notably, whether SQH can ameliorate diabetic myocardial injury, particularly whether it participates in regulating the ferroptosis process, which has garnered significant attention in recent years, has not been previously evaluated. In this study, we assessed the myocardial protective effects of SQH primarily by mitigating ferroptosis, a process evidenced by significant improvements in lipid peroxidation and mitochondrial function. Furthermore, we observed that SQH treatment also attenuated apoptosis *in vitro*, as indicated by reduced PI fluorescence intensity and cleaved caspase-3 expression (Supplementary Figure S1B, S1C). These findings indicate that SQH alleviates DbCM injury through a multimodal mechanism, with the inhibition of the ACSL4/ALOX15-mediated ferroptosis pathway representing the central mechanism.

The ACSL4/ALOX15 axis is a key enzymatic driver of lipid peroxidation, which is the central event in ferroptosis (Yang and Stockwell, 2016). Increasing evidence has shown the unique role of the ACSL4/ALOX15 axis in human diseases, such as hepatocellular carcinoma and ischemia/reperfusion (IR) injuries, but its significance in DbCM pathogenesis remains poorly understood (Ding et al., 2023). A recent study indicated that downregulation of ACSL4 expression ameliorated myocardial lipid accumulation and apoptosis in DbCM models (Wu et al., 2024). However, it elucidated only its role in lipid dysregulation, without examining the function of ACSL4-driven ferroptosis in this context. It has been proposed that lipid accumulation may affect mitochondrial function, which in turn indirectly suppresses ACSL4 translocation, thereby exacerbating mitochondria-associated ferroptosis in the development of DbCM (Cai et al., 2024; Zhao T. et al., 2025). Previous work by Cai et al. (2023) demonstrated that ALOX15 and its metabolites affect the expression of PGC-1α, leading to mitochondrial dysfunction in the IR model. Therefore, we assessed the expression of ACSL4 and its downstream effector ALOX15 at both the gene and protein levels and further demonstrated that the inhibition of the ACSL4/ALOX15 axis ameliorates mitochondrial dysfunction and myocardial injury by regulating PGC-1α protein expression in DbCM.

There is evidence that ferroptosis participates in the onset and progression of various cardiovascular diseases, including heart failure, myocardial infarction, and cardiomyopathy (Song et al., 2021; Wang et al., 2023; Cheng et al., 2024). As an iron-dependent form of regulated cell death driven by lipid peroxidation, ferroptosis has been increasingly implicated in the pathogenesis of DbCM (Li et al., 2025; Zhao Q. et al., 2025). Our findings showed that inhibiting the activated ACSL4/ALOX15–ferroptosis axis alleviated myocardial injury in DbCM, establishing ferroptosis suppression as a practical therapeutic target. Furthermore, our *in vitro* model revealed a positive association between the 12-HETE levels (metabolites of the ACSL4/ALOX15 axis in ferroptosis) and cardiomyocyte injury in DbCM, where the 12-HETE concentration increased with the degree of cellular damage (Supplementary Figure S1D-F). Future work will analyze the potential of 12-HETE as a diagnostic or prognostic biomarker for DbCM.

Our study established a foundation for further investigating SQH’s cardioprotective role and identifying a promising target for the early intervention of DbCM. However, several important questions remain to be fully elucidated. First, the precise mechanism by which SQH regulates ACSL4 expression is unclear; it may involve direct transcriptional control or indirect effects on its mitochondrial translocation, both of which warrant further investigation. Second, we observed biphasic modulation effects of SQH, with reduced efficacy at the high dose (400 mg/kg). This was consistent with many natural compounds (Wang et al., 2020). For instance, ginsenosides have demonstrated a similar biphasic effect in the adipose tissue of diet-induced diabetic models: they suppress TAG synthesis by increasing p-AMPK levels, yet they simultaneously promote adipogenesis via stimulation of the PPAR-γ pathway (Li and Ji, 2018). Thus, defining the optimal therapeutic window for SQH is crucial for its clinical application. Finally, ferroptosis inducers and inhibitors should be used in future experiments to further verify the role of SQH.

## 5 Conclusion

This study identified SQH as a potent cardioprotective agent that can effectively restore cell viability and ROS overload in H9c2 cells subjected to PA-induced lipotoxicity. We further demonstrated that ferroptosis serves as the predominant form of cell death in this model and found that SQH significantly attenuates ferroptosis-related manifestations, such as lipid peroxidation and mitochondrial dysfunction. Furthermore, SQH administration improved glucose homeostasis and cardiac function in a DbCM mouse model by regulating the ACSL4/ALOX15 pathway. These results highlighted that SQH could be considered a promising therapeutic candidate for the treatment of DbCM.

## Data Availability

The data presented in the study are deposited in the NCBI repository, accession number PRJNA1378467.
