# Supplementary material for: Panax notoginseng flower protects against diabetic cardiomyopathy by regulating the ACSL4/ALOX15 pathway
Source: Front Pharmacol. 2026 Mar 27;17:1780442. doi: 10.3389/fphar.2026.1780442 (PMC13066132; doi:10.3389/fphar.2026.1780442)
Supplement: Supplementary file 4 [file Supplementaryfile1.docx]

**Supplementary Figure and Table Captions**

Fig. S1 (A) Left ventricular fractional shortening (LVFS), Left ventricular ejection fraction (LVEF) and Left ventricular internal diameter at end-systole (LVIDs) were assessed *via* echocardiography. (B) Cell apoptosis detection, cells in early apoptotic stage emit green fluorescence, while cells in late apoptotic stage emit red and green fluorescence signals. (C) Protein expressions for cleaved-caspase3, Caspase3 in SQH treated H9c2 cells. (D) Cell viability under different PA concentrations. (E) 12-HETE level of H9c2 cell with SQH treatment under 0.1 mM PA. (F) 12-HETE level of H9c2 cell under different PA concentrations.

Table S1 Primer sequences for the genes used for qRT-PCR.

Table S2 The results of KEGG terms enrichment analysis in the H9c2 cells.

Table S3 FPKM values of H9c2 cells.

Table S4 Differentially expressed genes related to ferroptosis of H9c2 cells.

Table S5 Original WB blots.
