# Supplementary figures and images for: Panax notoginseng flower protects against diabetic cardiomyopathy by regulating the ACSL4/ALOX15 pathway
Source: Front Pharmacol. 2026 Mar 27;17:1780442. doi: 10.3389/fphar.2026.1780442 (PMC13066132; doi:10.3389/fphar.2026.1780442)

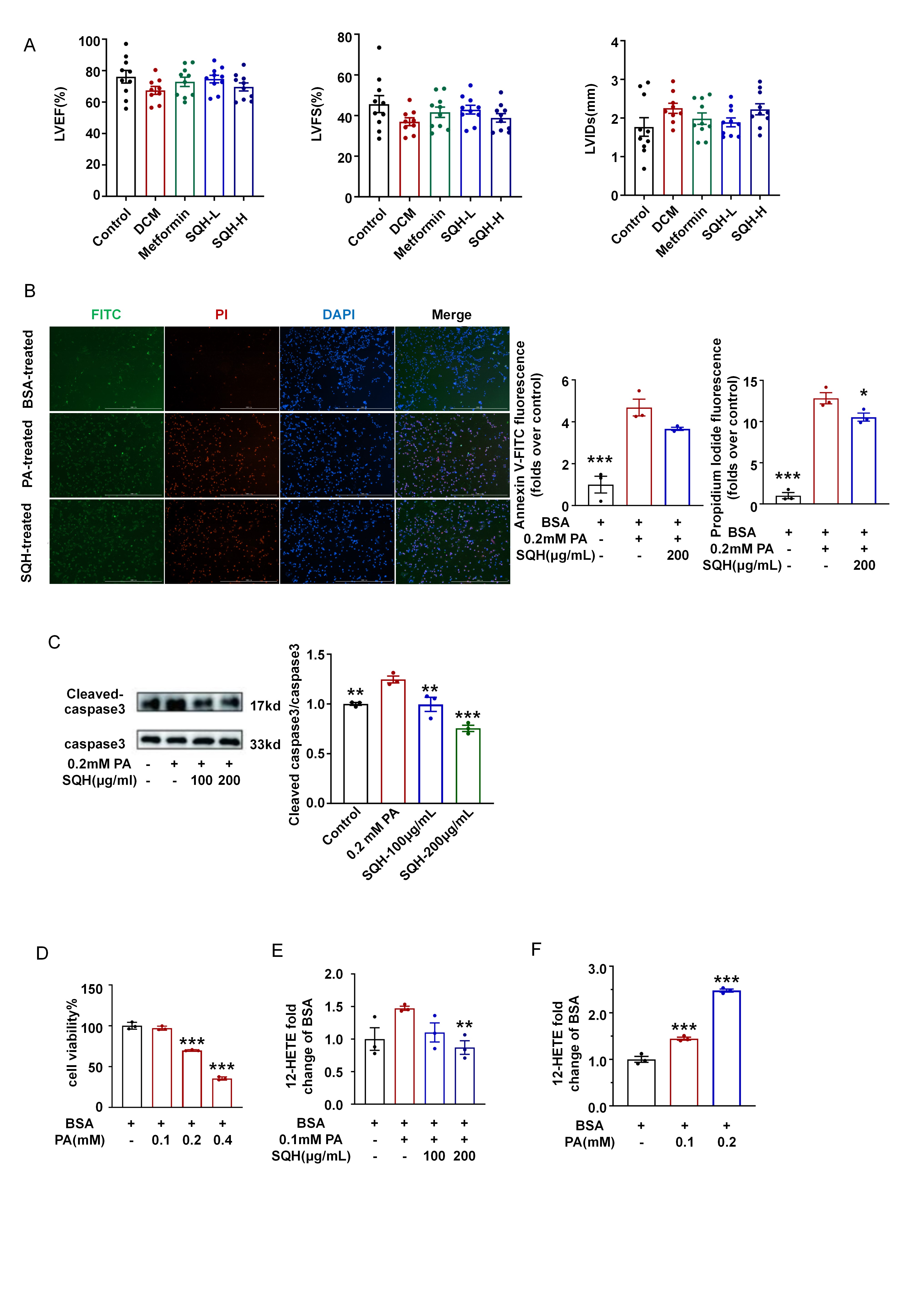

Supplement: Supplementary file 5 [file Image1.jpeg]
