# Supplementary material for: Panax notoginseng flower protects against diabetic cardiomyopathy by regulating the ACSL4/ALOX15 pathway
Source: Front Pharmacol. 2026 Mar 27;17:1780442. doi: 10.3389/fphar.2026.1780442 (PMC13066132; doi:10.3389/fphar.2026.1780442)
Supplement: Supplementary file 8 [file DataSheet1.docx]

**Supplementary Figure and Table Captions for**

**Panax notoginseng flower protects against diabetic cardiomyopathy via regulating ACSL4/ALOX15 pathway**

| ALOX15 75Kd | β-actin 45Kd |
| --- | --- |
| 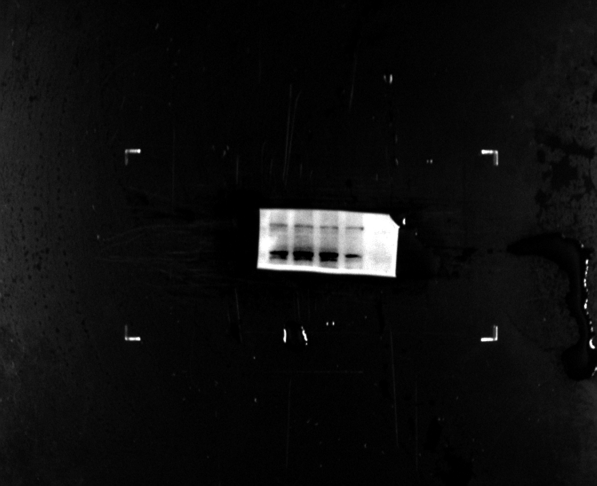 | 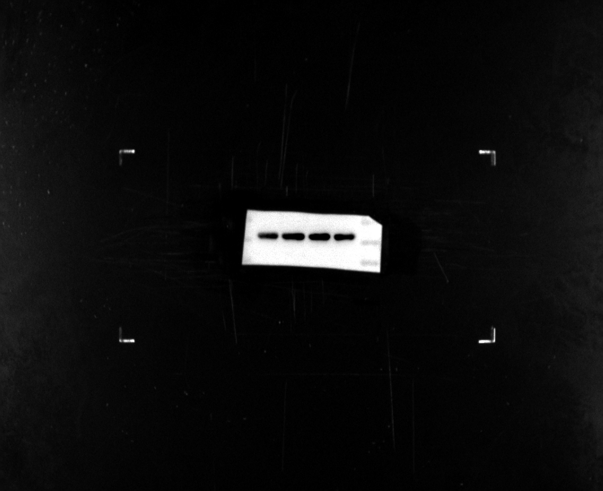 |
| 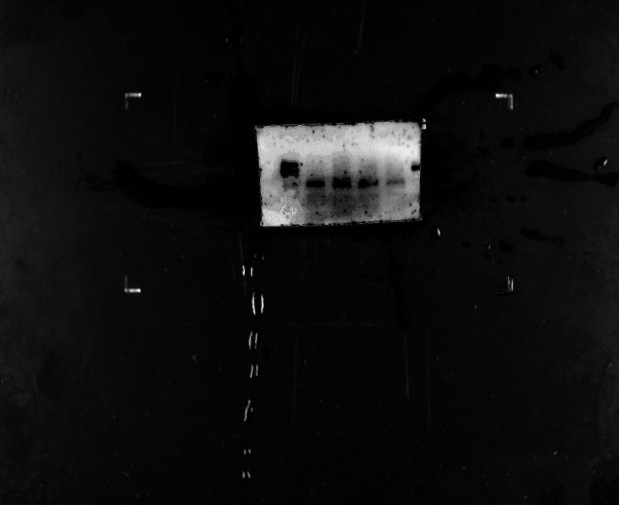 | 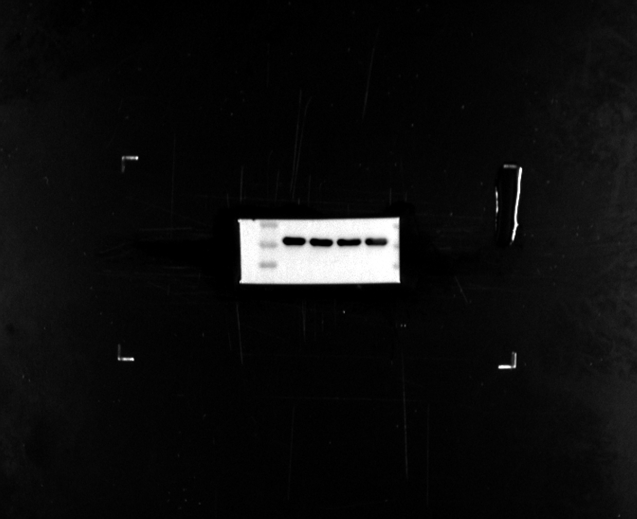 |
| 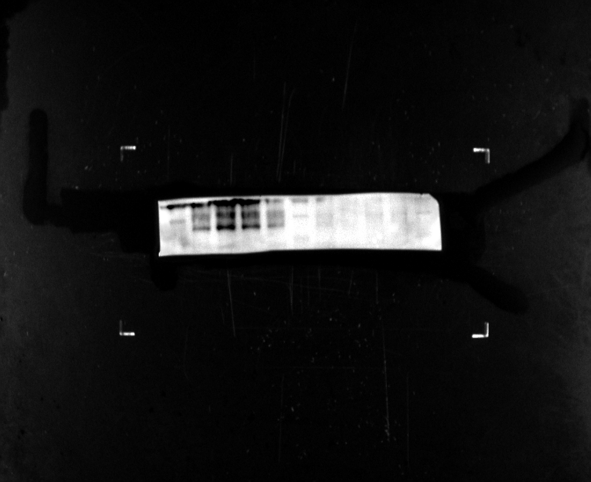 | 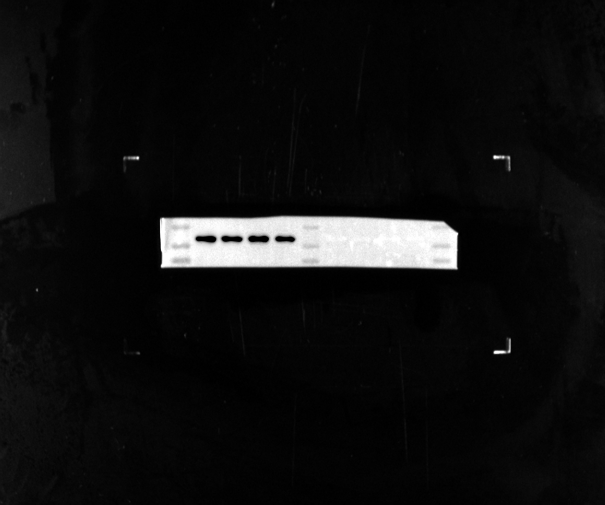 |
| 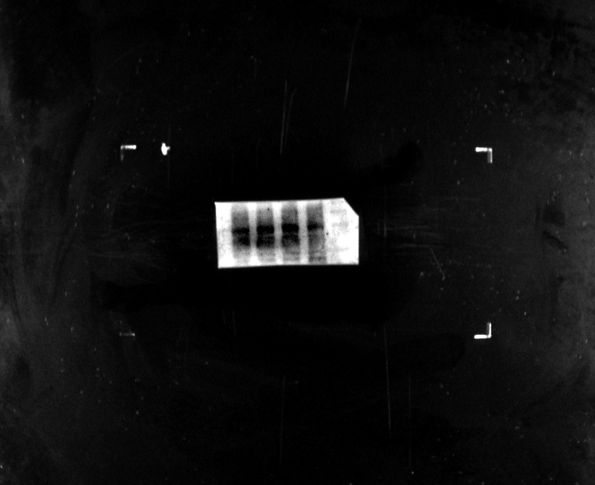 | 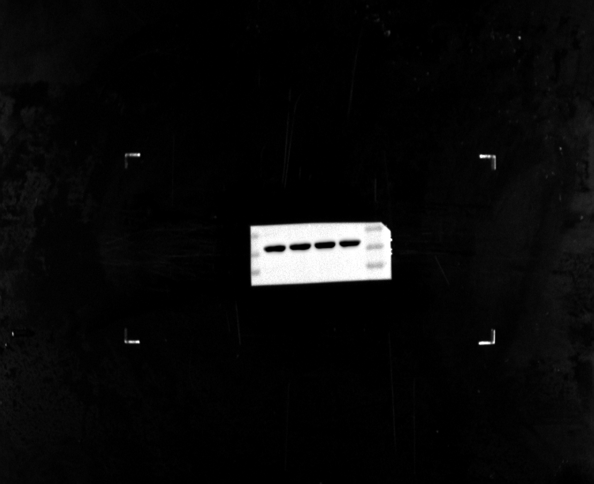 |

| ACSL4 79Kd | β-actin 45Kd |
| --- | --- |
| 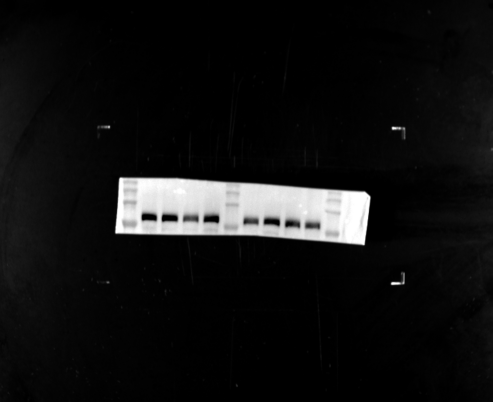 | 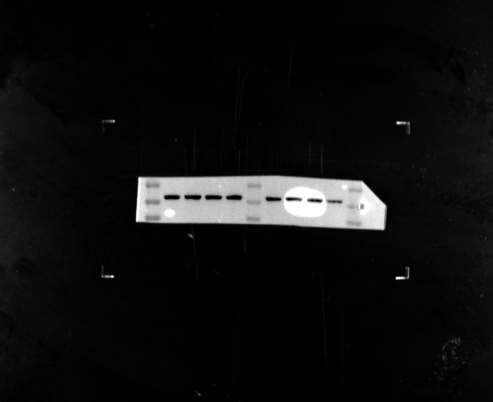 |
| 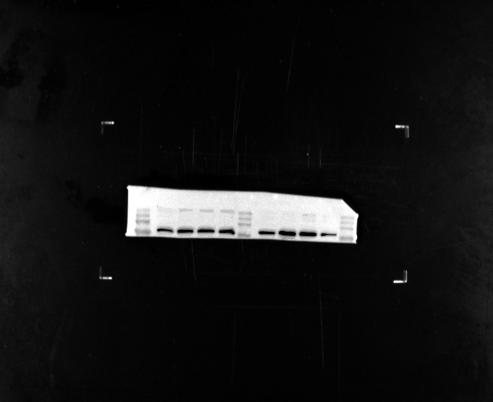 | 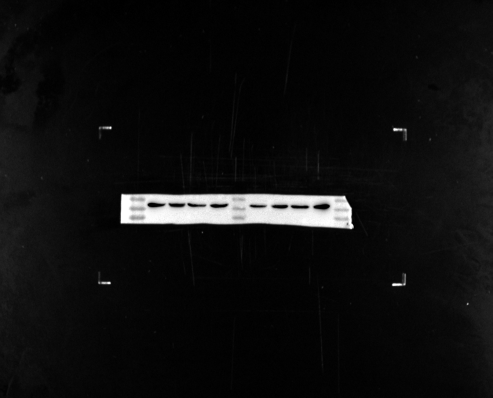 |
| 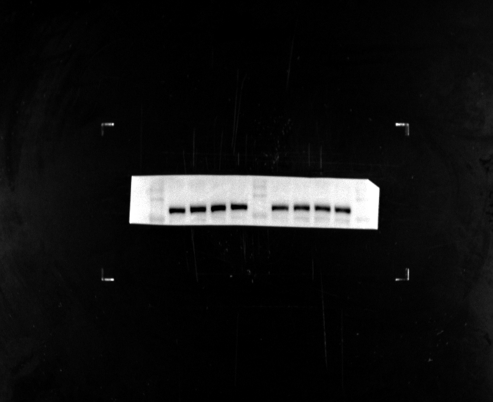 | 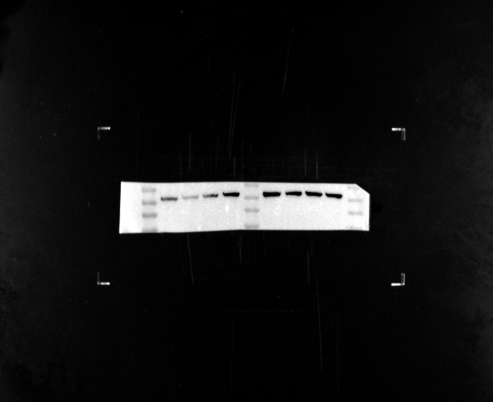 |

| GPX4 22Kd | β-actin 45Kd |
| --- | --- |
| 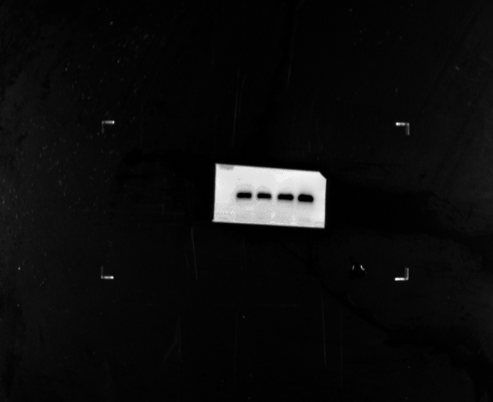 | 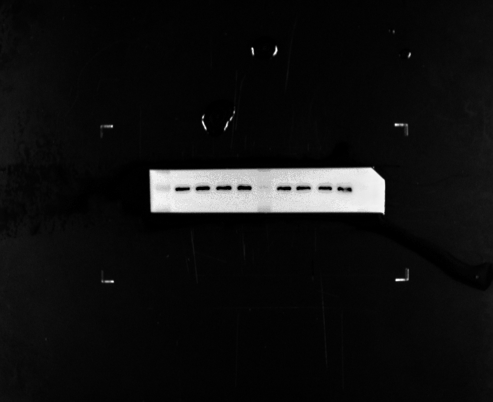 |
| 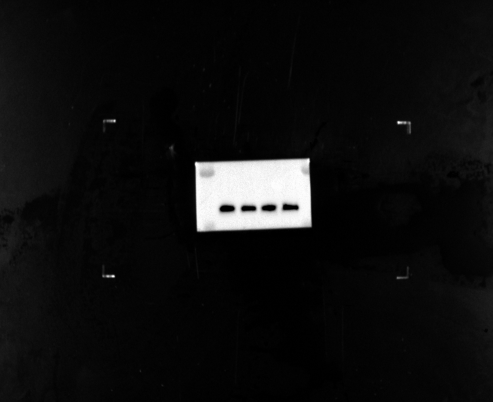 | 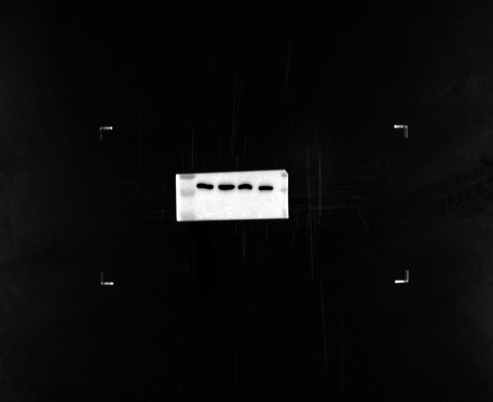 |
| 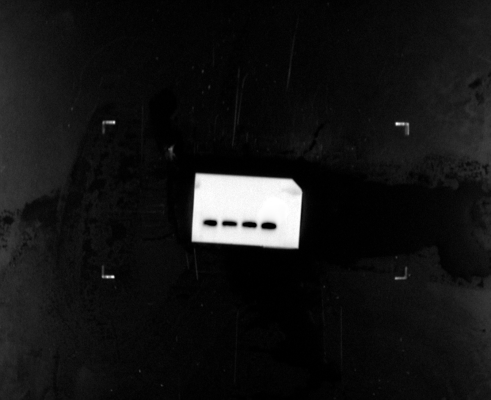 | 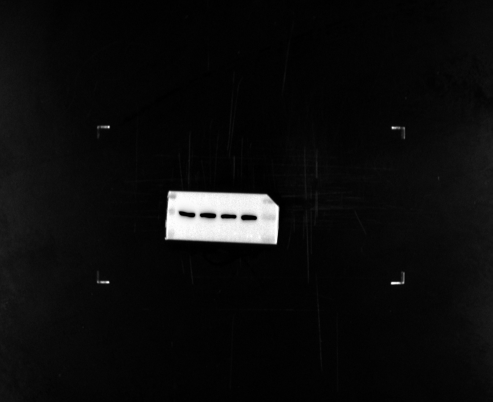 |
| 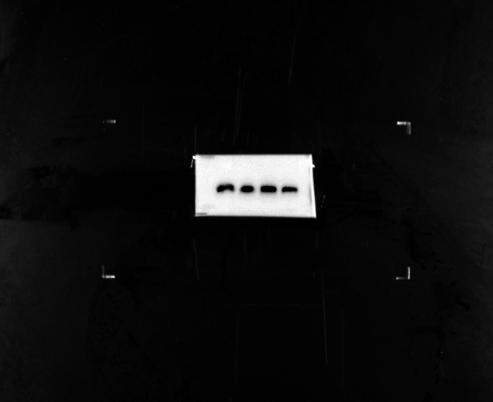 | 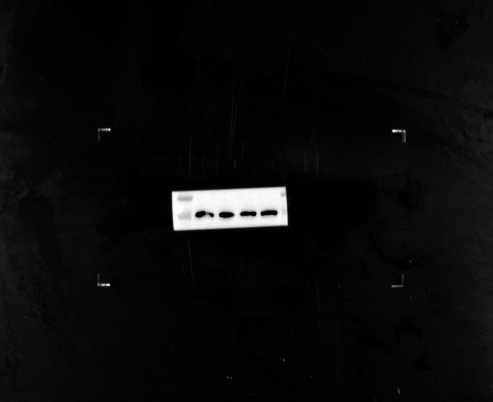 |
| 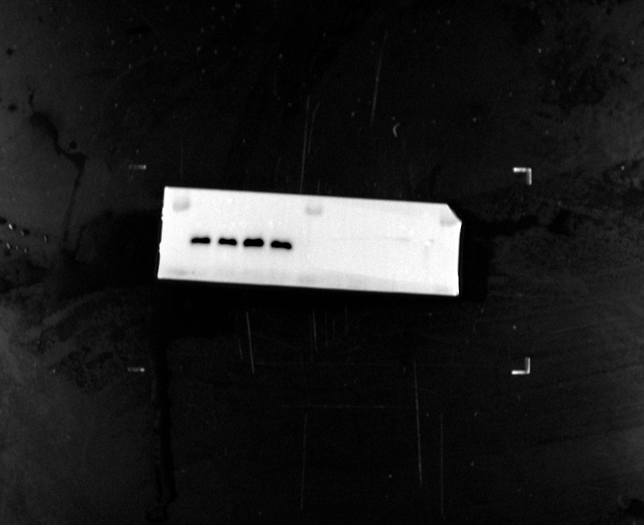 | 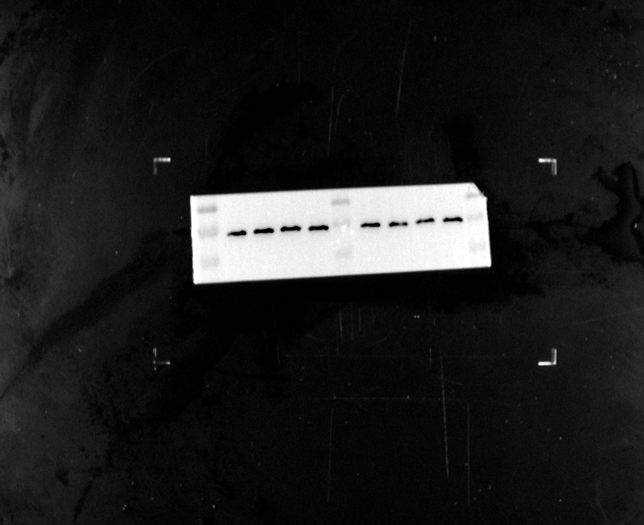 |

| Cytochrome c 12Kd | β-actin 45Kd |
| --- | --- |
| 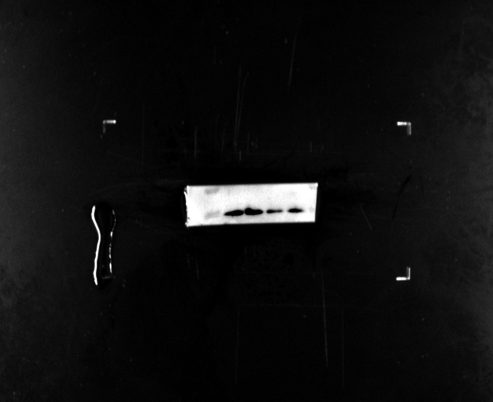 | 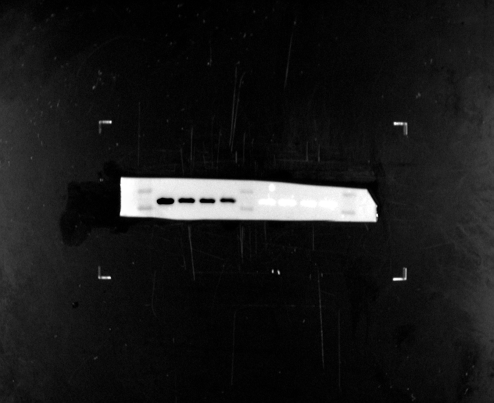 |
| 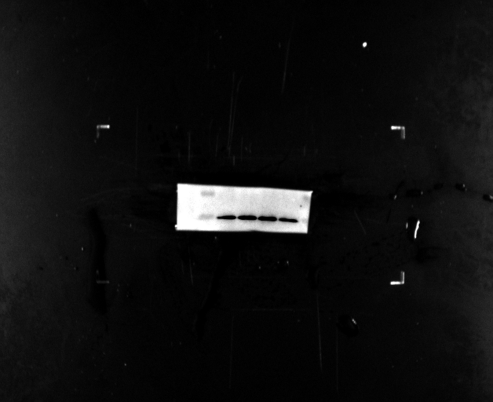 | 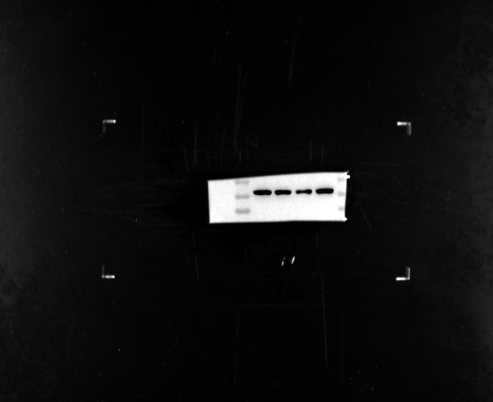 |
| 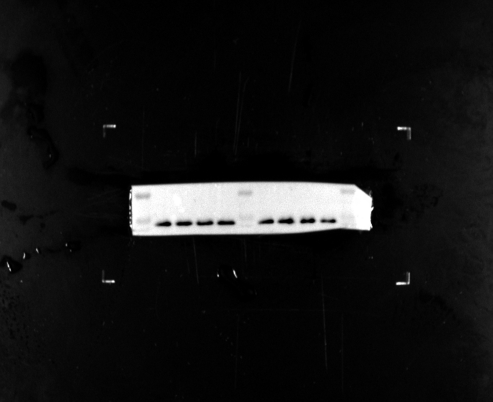 | 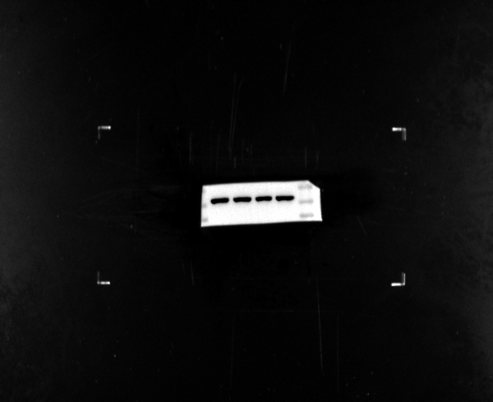 |

| Cleaved caspase3 12Kd | Caspase3 32Kd |
| --- | --- |
| 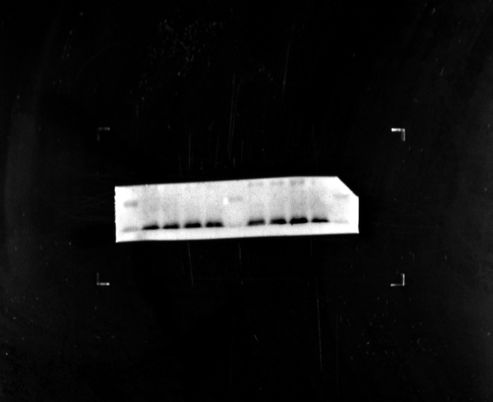 | 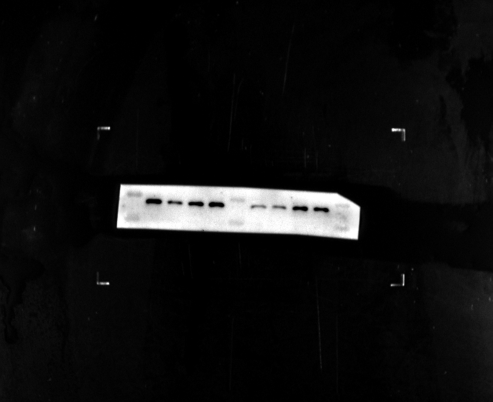 |
| 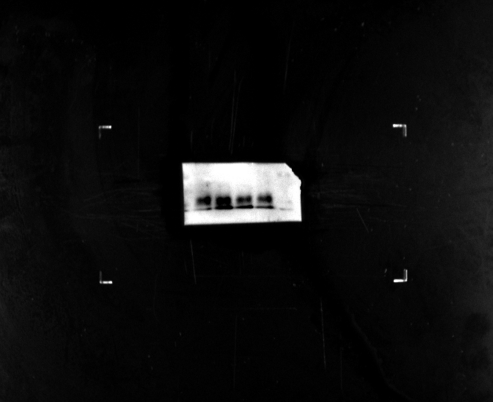 | 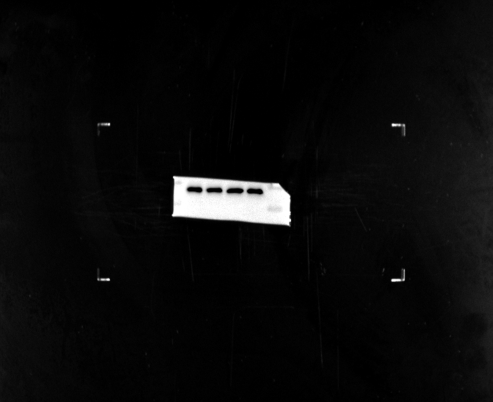 |
| 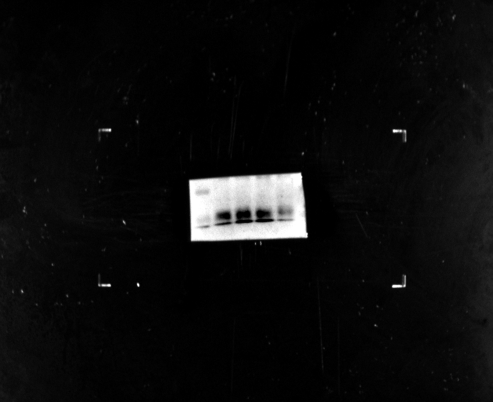 | 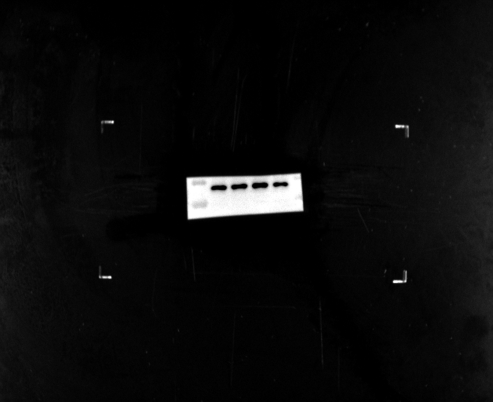 |

| PGC-1α 91Kd | β-actin 45Kd |
| --- | --- |
| 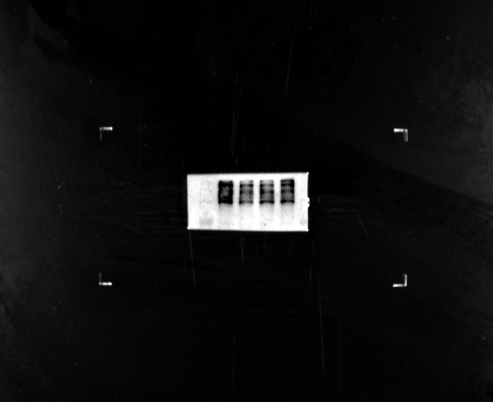 | 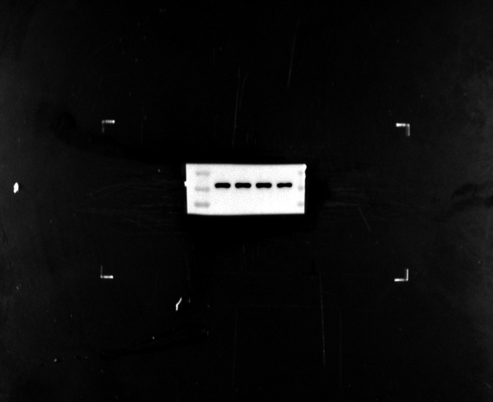 |
| 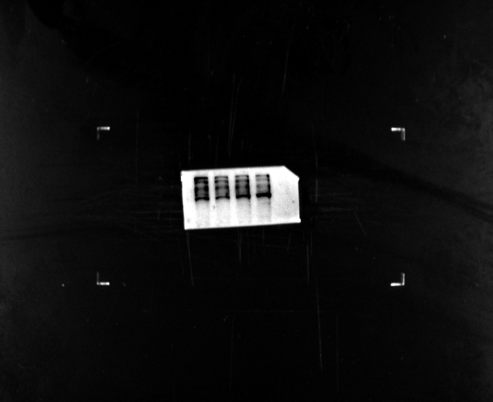 | 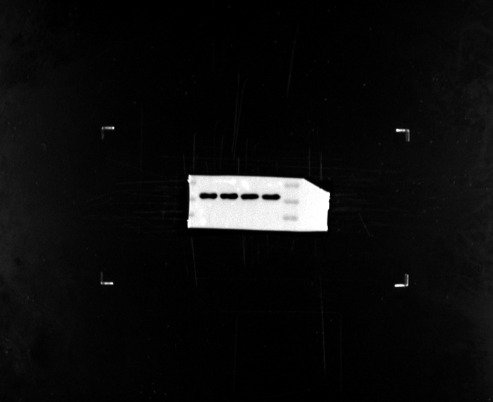 |
| 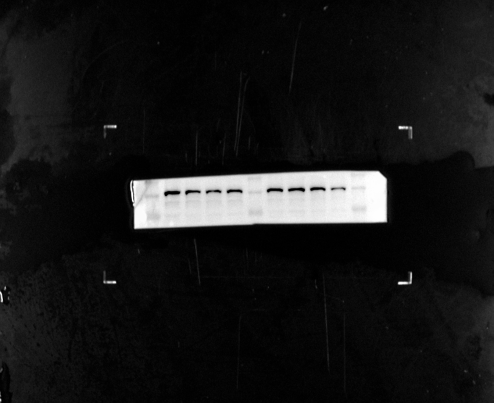 | 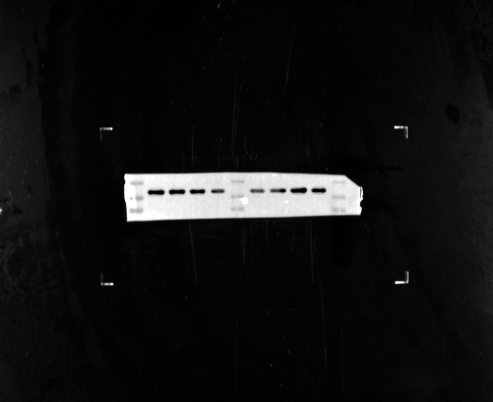 |

| Protein of animal tissue |
| --- |
| **ALOX15**  **75kd**  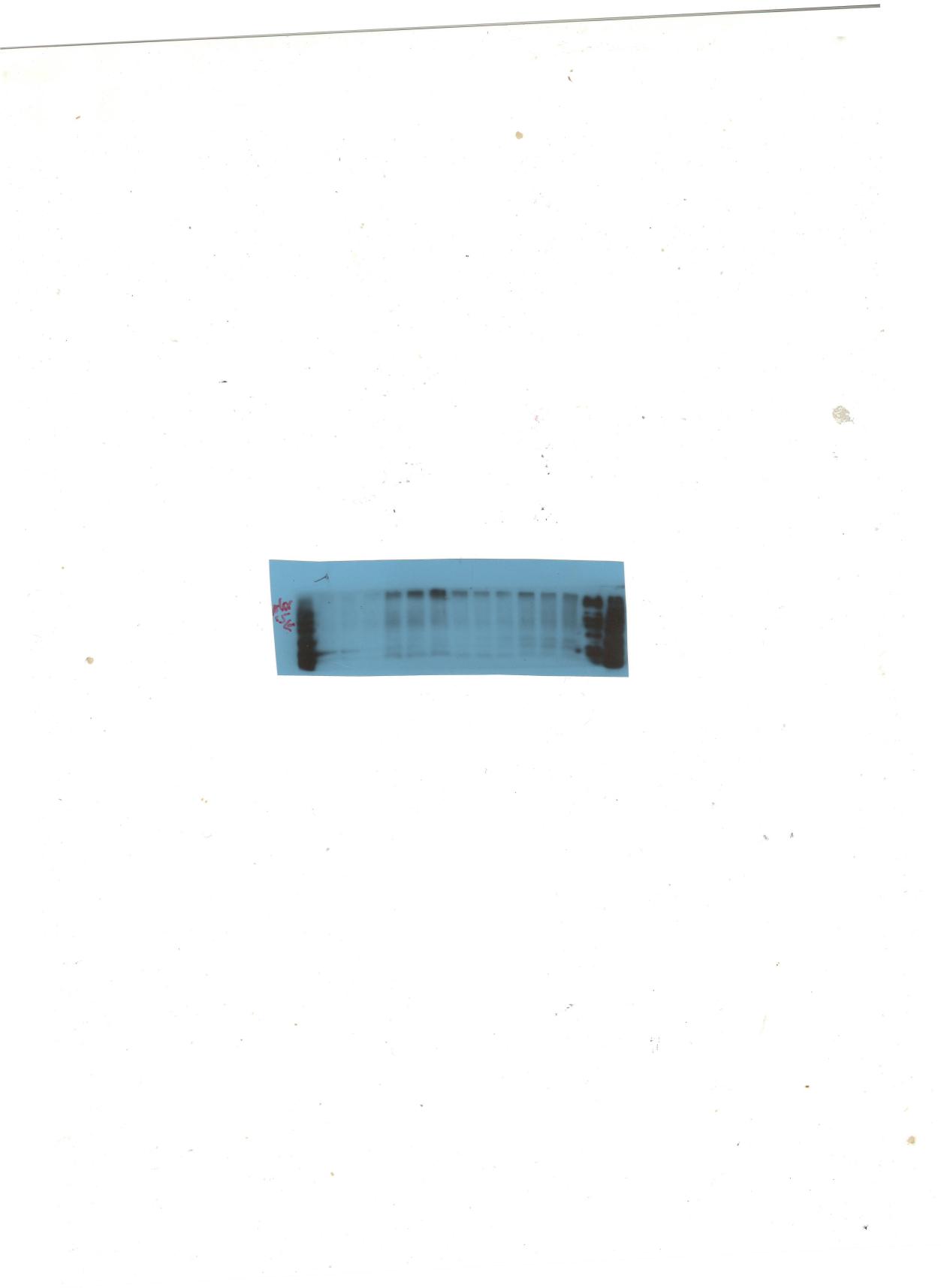  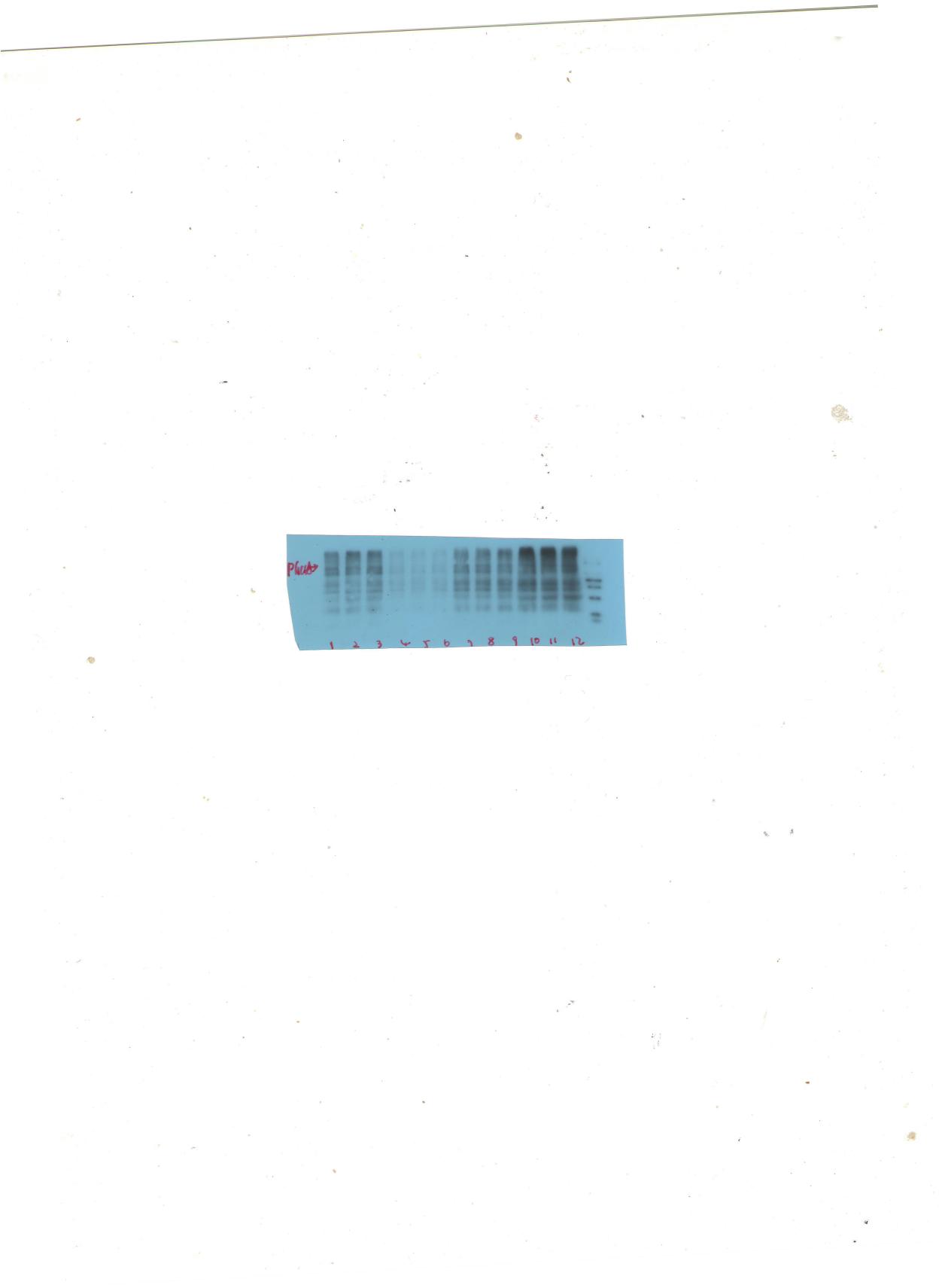  **PGC-1α**  **91kd**  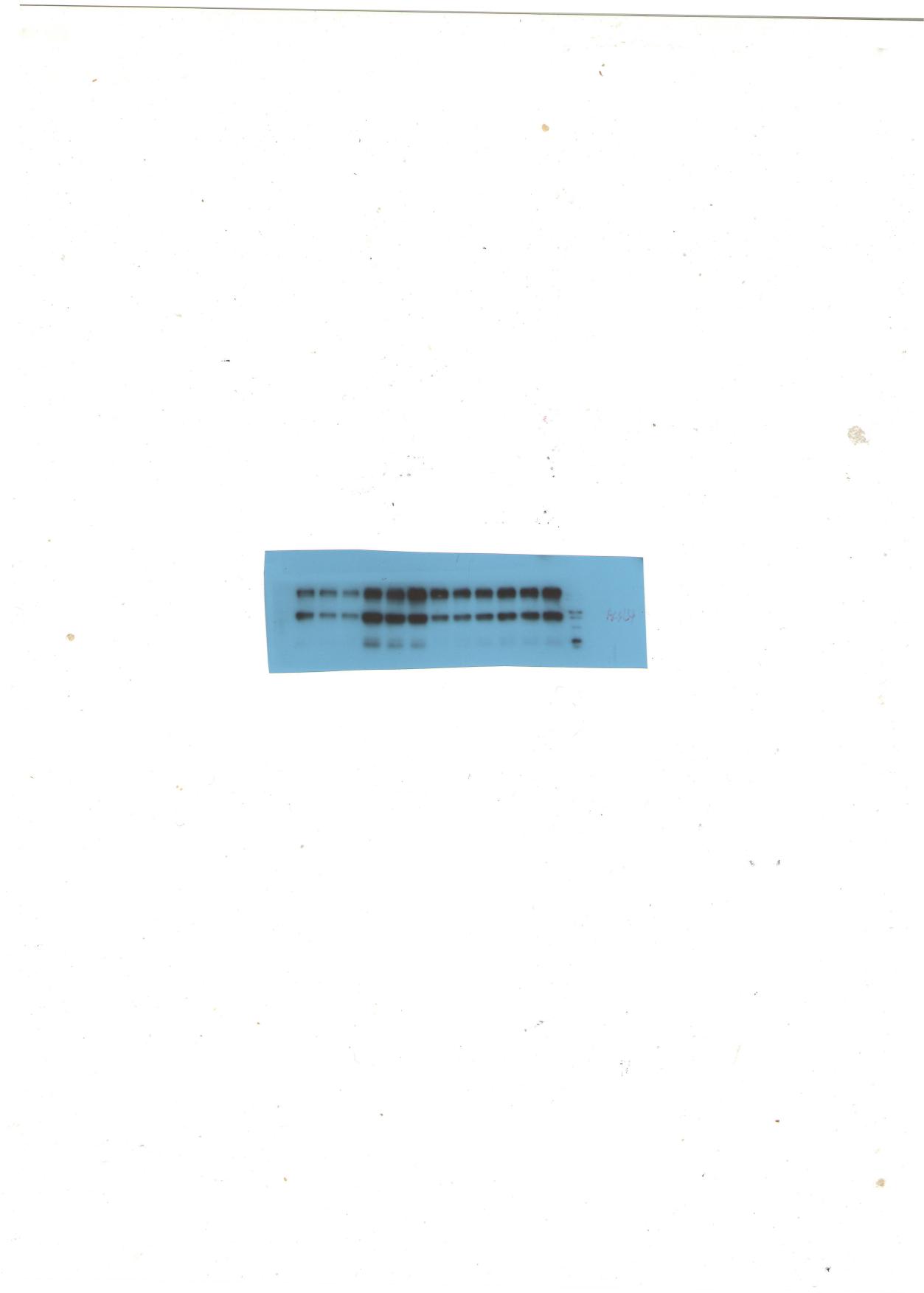  **ACSL4**  **79kd**  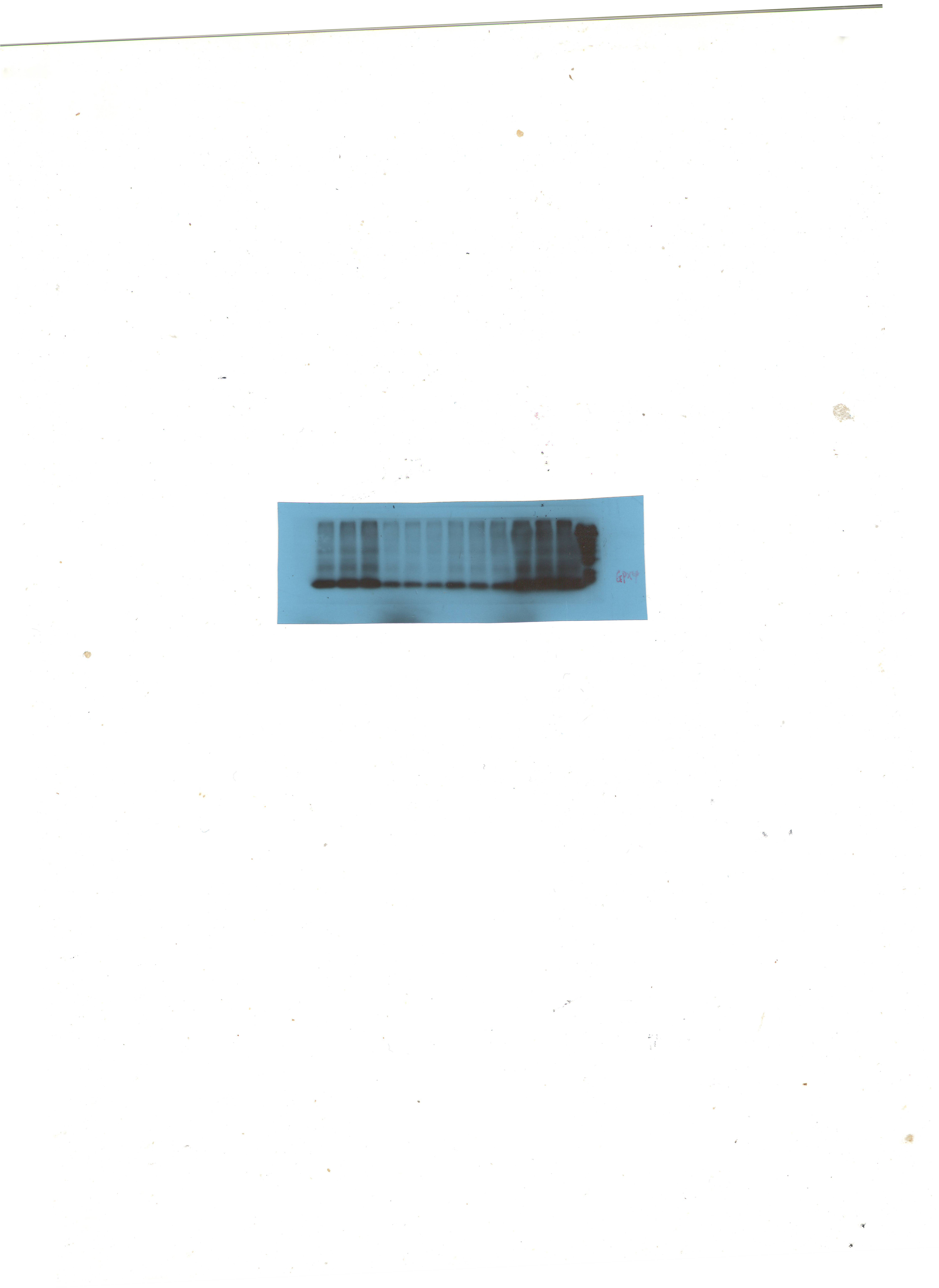  **GPX4**  **22kd**  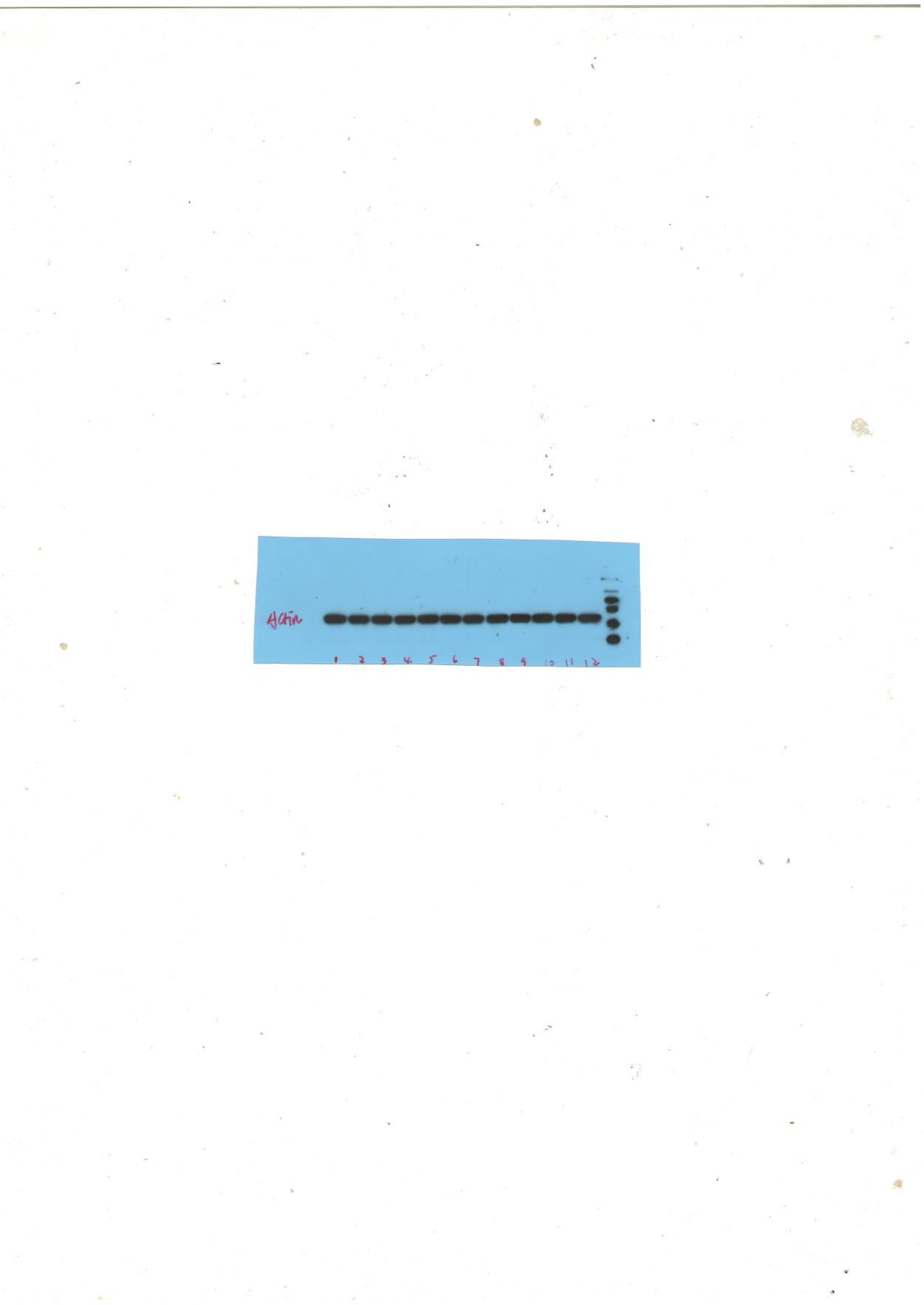  **ACTIN 45kd** |
